# Interactions of the Tricyclic Antidepressant Drug Amitriptyline with L-DOPA in the Nucleus Accumbens, Prefrontal Cortex and Hippocampus of Unilaterally 6-OHDA-Lesioned Rats: Relevance to Depression in Parkinson’s Disease

**DOI:** 10.3390/biom16050743

**Published:** 2026-05-19

**Authors:** Kinga Kamińska, Tomasz Lenda, Jolanta Konieczny, Elżbieta Lorenc-Koci

**Affiliations:** Maj Institute of Pharmacology, Polish Academy of Sciences, Smętna Street 12, 31-343 Kraków, Poland; k.kamin@if-pan.krakow.pl (K.K.);

**Keywords:** amitriptyline, L-DOPA, depression, Parkinson’s disease, 6-OHDA, lesion, nucleus accumbens, prefrontal cortex, hippocampus

## Abstract

The effects of antidepressants on limbic structures, important in the context of the treatment of Parkinson’s disease (PD)-associated depression, are relatively poorly explored in animal models. The present study investigated the impact of the tricyclic antidepressant amitriptyline (AMI), administered chronically alone or in combination with L-DOPA, on anhedonia, monoamine levels, and the binding of radioligands to their transporters in the limbic structures of unilaterally 6-OHDA-lesioned rats. Anhedonia, as a core symptom of depression, was evaluated using the sucrose preference test. Tissue concentrations of noradrenaline (NA), dopamine (DA) and serotonin (5-HT) and their metabolites in the prefrontal cortex (PFC) and hippocampus (HIP) were assayed by HPLC method. Bindings of [^3^H]nisoxetine to noradrenaline transporter (NET), [^3^H]GBR 12,935 to dopamine transporter (DAT), and [^3^H]citalopram to serotonin transporter (SERT) in the nucleus accumbens (NAcc), PFC, and HIP were analyzed by autoradiography. Three weeks of treatment of unilaterally 6-OHDA-lesioned rats with AMI alone significantly reduced the intake of sucrose solution compared to the sham-operated control, but the combined administration of AMI+L-DOPA enhanced sucrose consumption. Administration of AMI+L-DOPA increased tissue DA concentrations in the lesioned and intact PFC and HIP more distinctly than L-DOPA alone. L-DOPA alone significantly decreased tissue 5-HT content in the lesioned PFC and HIP, while the addition of AMI reversed this effect. 6-OHDA administered unilaterally into the MFB drastically decreased DAT binding in the lesioned NAcc while increasing it on the intact side. Neither AMI nor L-DOPA, given alone or jointly, affected DAT binding in the lesioned NAcc. SERT binding was significantly reduced in the PFC, NAcc and HIP on both sides of the brain in the AMI- or AMI+L-DOPA-treated groups. NET binding decreased in the PFC and NAcc in the AMI-treated group, but no such effect was observed in the AMI+L-DOPA-treated group. The obtained results are discussed in relation to the impaired psychiatric functions in PD.

## 1. Introduction

Parkinson’s disease (PD) is a neurodegenerative disorder that develops gradually over a long period, ultimately leading to the appearance of characteristic motor symptoms in advanced age, such as bradykinesia, rigidity, tremor, and postural instability [1]. In addition to motor impairments, patients with PD suffer from numerous non-motor symptoms, the manifestation of which usually precedes the motor disturbances [2,3,4]. Non-motor symptoms include neuropsychiatric disorders (e.g., depression, anxiety, apathy, psychosis), autonomic dysfunction, fatigue, and sleep problems [1,5,6,7,8,9,10]. Furthermore, non-motor symptoms are frequently even more debilitating than the motor symptoms, significantly worsening the quality of life of patients with PD. However, they often remain unrecognized by physicians and, therefore, untreated.

Initially, PD was considered a purely motor disorder due to the selective degeneration of dopaminergic neurons in the substantia nigra pars compacta (SNc). However, more recent neuropathological data demonstrating neurodegenerative processes in other monoaminergic systems [11,12,13,14,15] and the high incidence of non-motor disturbances [5,10,16,17] have led to its more precise definition as a neuropsychiatric disorder [18]. Among non-motor symptoms, depression is an important neuropsychiatric symptom accompanying PD that occurs in an average of 24–50% of patients [10,19,20,21,22,23,24,25]. Its incidence may increase to almost 90% [18,26,27].

Depression appears already in the pre-motor prodromal phase of PD and continues to be present in its later stages, varying in symptom severity depending on the motor state. It appears to be unrelated to age but is related to the progression of the disease [21,23]. Hence, it is believed that depression in PD is a secondary response to the manifestation of neurodegenerative changes rather than a reaction to psychosocial stress. In comparison to depression observed in patients without PD, its course is milder, but anhedonia and apathy are more common [28]. The diagnosis of depression in patients with PD in the advanced stage is complicated because the presence of motor deficits, such as psychomotor slowing and reduced facial expression, may interfere with the ability to evaluate the characteristic symptoms of depression that overlap with them.

The degenerative processes in PD include loss of noradrenergic, serotonergic, and dopaminergic neurons in the locus coeruleus (LC), raphe nucleus (RN), substantia nigra (SN), and ventral tegmental area (VTA), respectively [11,12,13,14,15]. Dysfunction of the dopaminergic nigrostriatal pathway is responsible for motor impairment in PD. Anhedonia, a core symptom of depression, defined as a lowered ability to experience physical or social pleasure observed in a major depressive disorder, is frequent in depressed PD patients [22,29,30,31,32]. It is believed to be caused by the impairment of dopaminergic reward mechanisms, which are closely linked to the degeneration of the VTA and limbic projections [33]. In depressed PD patients, the limbic noradrenergic/dopaminergic pathways are dysfunctional compared to those without depression [34]. Regarding dysfunction in the serotonergic system, PD patients with depression had greater neuronal loss in the dorsal raphe nuclei (DRN) than non-depressed PD patients [14]. Moreover, serotonergic transmission was found to be decreased in the basal ganglia, including the caudate nucleus, putamen, globus pallidus (GP), and SN, as well as in the thalamus [35,36,37], frontal, cingulate, and enthorinal cortices (by 40–60%), and in the hippocampus [38,39].

In our previous study, a neurochemical model of symptomatic PD with coexisting depressive-like symptoms and motor impairment was developed [40]. This model was based on the application of the classic neurotoxin 6-hydroxydopamine (6-OHDA), which is commonly used to study pathomechanisms and potential therapeutic strategies in PD [41,42]. In our model, administration of 6-OHDA at a dose of 16 µg/4 µL unilaterally into the medial forebrain bundle (MFB), without prior administration of desipramine (DES), which is often used to protect noradrenergic terminals, caused drastic decreases in the DA concentration in the striatum (STR), SN, hippocampus (HIP) and prefrontal cortex (PFC) on the lesioned side [40]. In all these structures, except for the SN, a very potent decrease in noradrenaline (NA) and ca. 50–60% drop in serotonin (5-HT) contents were also found on this side. These changes in monoamine levels in rats with unilateral 6-OHDA lesion corresponded well with similar changes in their content seen in PD patients [35,36,37,38]. Furthermore, these model rats showed deficits in motor function and depressive-like behavior measured in appropriate tests [40]. Additionally, in this model of PD, an analysis of monoamine metabolism and radioligand binding to their transporters in the STR and SN was performed in groups of rats administered the tricyclic antidepressant drug amitriptyline (AMI) and antiparkinsonian drug L-DOPA, either alone or in combination, in the context of motor performance [43]. This study showed that chronic administration of AMI+L-DOPA more strongly increased tissue DA concentrations in the lesioned STR and SN than L-DOPA alone, resulting in a greater improvement in motor function after combined treatment than after L-DOPA alone, as assessed by rotational behavior [43]. However, the impact of these drugs on depressive-like behavior has not been examined yet. Therefore, in the present study, we decided to perform the same analysis of monoamine metabolism and radioligand binding to their transporters in the chosen limbic brain structures, i.e., the nucleus accumbens (NAcc) core and shell, hippocampus (HIP), and prefrontal cortex (PFC). AMI was administered at a dose of 10 mg/kg, which is commonly used to treat depressive-like behavior in rodents [44]. L-DOPA was administered at a dose of 12 mg/kg, which effectively increased the concentration of the functional, synaptic DA pool in the STR and SN in unilaterally 6-OHDA-lesioned rats [45]. Moreover, this dose of L-DOPA also effectively improves motor function, assessed as rotational behavior [43]. Lower doses of L-DOPA, 3 or 6 mg/kg, are insufficient to induce asymmetric behavior in unilateral 6-OHDA-lesioned rats and are therefore used to model dyskinesia. Higher doses of L-DOPA (25 mg/kg), however, induce excessive rotational behavior. We hope that these experiments will contribute to a better understanding of the mechanisms underlying depression in PD and will allow us to assess the effectiveness of the combined therapy with AMI+L-DOPA on psychiatric symptoms.

## 2. Materials and Methods

The experiments were carried out in a compliance with the Act on the Protection of Animals Used for Scientific or Educational Purposes of 21 January 2005, reapproved on 15 January 2015 (published in Journal of Laws no 23/2015 item 266, Poland), and according to the Directive of the European Parliament and of the Council of Europe 2010/63/EU of 22 September 2010 on the protection of animals. The Local Ethics Committee of the Maj Institute of Pharmacology, Polish Academy of Sciences, in Kraków, Poland (permission no. 709/2010 of 28 January 2010) also approved the protocols for the planned experiments. Every effort was made to minimize the number of rats used in the particular experiments to the minimum necessary to ensure statistically reliable results (3R policy), and to limit their suffering and distress.

### 2.1. Animals

The study was conducted on male Wistar Han rats (Charles River, Sulzfeld, Germany) with an initial body weight of 250–275 g (for the sucrose pretest onset) or 290–320 g (for stereotaxic surgery), respectively, kept in standard cages measuring 590 mm long × 380 mm wide × 200 mm high, 5 animals per cage, with unlimited access to drinking water and standard laboratory food. The animals were provided with standard housing conditions, i.e., an artificial 12 h light/dark cycle (7:00–19:00 light, 19:00–7:00 dark), temperature (22 °C), and humidity (40–60%). The animals were experimentally naive.

### 2.2. Drugs

Amitriptyline hydrochloride (AMI; Cat No. A8404), apomorphine hydrochloride (Cat. No. A43930), benserazide hydrochloride (Cat. No. B7283), 3,4-dihydroxy-L-phenylalanine methyl ester (L-DOPA; Cat. No. D1507), 6-hydroxydopamine hydrochloride (6-OHDA; Cat. No. H4381) and L-ascorbic acid (Cat. No. A0278), as well as standards for noradrenaline (NA; Cat. No. 74480), dopamine (DA; Cat. No. H8502), 3,4-dihydroxyphenylacetic acid (DOPAC; Cat. No. 37860), 3-methoxytyramine (3-MT; Cat. No. M4251), homovanillic acid (HVA; Cat. No. H1252), serotonin (5-HT; Cat. No. 85036) and 5-hydroxyindoleacetic acid (5-HIAA; Cat. No. H8876), were provided by the Sigma-Aldrich Chemical Company (Steinheim, Germany), while bovine serum albumin (Cat. No. A7906), cis-flupentixol (Cat. No. BP738), mazindol (Cat. No. M2017), and fluoxetine (Cat. No. F132) were provided by the Sigma-Aldrich Chemical Company (St. Louis, MO, USA). The labeled ligands for binding studies to monoamine transporters, i.e., to dopamine transporter (DAT)—[^3^H]1-[2-(diphenyl-methoxy)-ethyl]-4-(3-phenylpropyl)piperazine ([^3^H]GBR 12,935), to serotonin transporter (SERT)—[^3^H]citalopram, and to noradrenaline transporter (NET)—[^3^H]nisoxetine, were provided by Perkin Elmer Life and Analytical Sciences (Boston, MA, USA). Ketamine and diazepam were supplied by Biowet (Sp. z o.o., Puławy, Poland) and Polfa (Warszawa, Poland), respectively.

### 2.3. Experimental Design

**Figure 1:**
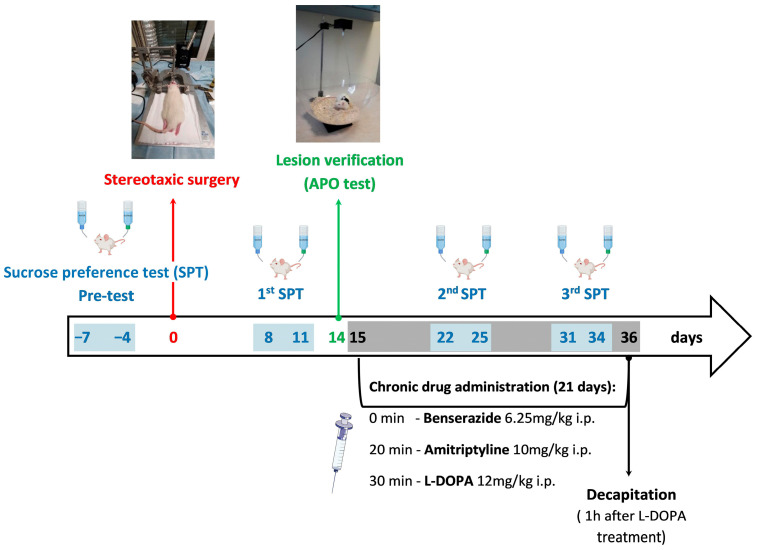
The schedule of the performed experimental procedures, including behavioral tests (sucrose preference test, SPT; rotational behavior, apomorphine test), stereotactic surgeries (unilateral administration of a single dose of 6-OHDA into the MFB without desipramine pretreatment), and chronic administration of the studied drugs.

The behavioral and biochemical interactions of the tricyclic antidepressant drug AMI with L-DOPA were examined in rats with unilateral 6-OHDA-induced lesion of the dopaminergic, noradrenergic, and serotonergic pathways, which in our previous study was proposed as a model of symptomatic PD with the coexisting depression-like behavior [40]. Control rats, instead of the unilateral 6-OHDA administration into the medial forebrain bundle (MFB), received the vehicle. The time-course diagram of the experiments performed is presented in Figure 1.

The sucrose preference test was performed 1 week before, as well as 1, 3, and 4.5 weeks after, stereotaxic surgery. Two weeks (13th day) after unilateral 6-OHDA injection into the MFB, these rats were tested for the rotational behavior induced by APO (0.25 mg/kg s.c.). On the next day, rats exhibiting more than 100 contralateral turns/1 h in this test, which, according to our previous studies [40,46,47], indicated the extensive unilateral lesion of the nigrostriatal dopaminergic system, were randomly divided into groups treated once daily with AMI (10 mg/kg i.p.) and L-DOPA (12 mg/kg i.p.), either alone or in combination, for 21 consecutive days. Rats were randomly allocated to control and treatment groups using simple random assignment. No formal randomization sequence generation or statistical randomization software was applied prior to allocation. The investigators were not blinded to the experimental conditions. No specific measures were implemented to control for potential confounding factors, including treatment order, measurement sequence, or animal/cage location.

**Figure 2:**
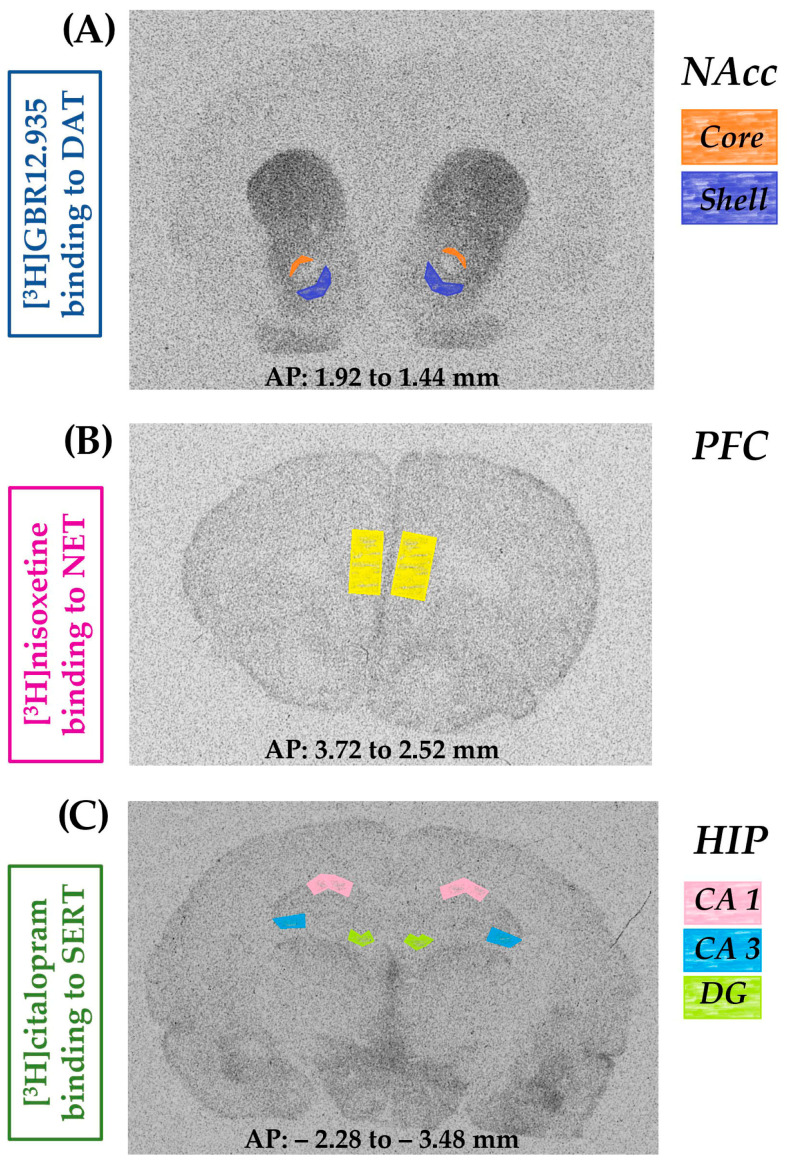
Representative autoradiograms of coronal brain sections. Individual limbic structures are marked on these sections: (**A**)—nucleus accumbens (NAcc; core, shell); (**B**)—prefrontal cortex (PFC); (**C**)—hippocampus (HIP; regions CA1, CA2, and dentate gyrus, DG). To illustrate which brain sections were used to analyze radioligand binding to monoamine transporters (NET, DAT, SERT), the stereotaxic anterior–posterior (AP) coordinates relative to the bregma are provided below each of these structures, according to the Paxinos and Watson atlas [48].

Benserazide hydrochloride (6.25 mg/kg i.p.) was injected 30 min before L-DOPA. Throughout the text, the combined administration of benserazide + L-DOPA was referred to as L-DOPA treatment. Benserazide hydrochloride and AMI were dissolved in redistilled water, L-DOPA methyl ester in 0.9% NaCl, and all of these compounds were injected at a volume of 1 mL/kg. One hour after the last doses of these drugs were given, rats were killed by decapitation, and their brains were isolated. The brains from all experimental groups were divided into two sets, designated either for high-performance liquid chromatography (HPLC) analyses of monoamine levels or for autoradiographic analysis of the binding of labeled ligands to monoamine transporters in the selected brain structures. To determine monoamine concentrations in the prefrontal cortex (PFC) and hippocampus (HIP) in all experimental groups, the lesioned and intact sides of each structure were separately isolated from the whole brain on a chilled plate and then immediately frozen on dry ice and stored at −80 °C until neurochemical quantification. For the autoradiographic study, the whole brains were frozen in cold heptane on dry ice and stored at −80 °C. The autoradiographic analysis of the labeled ligand binding to dopamine transporter (DAT), serotonin transporter (SERT), and noradrenaline transporter (NET) was performed on coronal brain sections encompassing the core and shell of the NAcc, the hippocampal regions CA1, CA3, and the dentate gyrus (DG), and the PFC. Figure 2 shows coronal sections of the rat brain with the marked limbic structures (NAcc, PFC, HIP), where the binding of labeled ligands to monoamine transporters (NET, DAT, SERT) was analyzed.

### 2.4. Stereotaxic Surgery

This procedure followed the previously described method [40,43]. Briefly, rats scheduled for surgery were lightly anesthetized with ketamine (50 mg/kg) and diazepam (2.5 mg/kg). Both drugs were administered intraperitoneally (i.p.) as a 1:1 mixture (*v*/*v*) in a volume of 1 mL/kg of body weight. Each anesthetized rat was then placed in a stereotaxic apparatus (David Kopf Instruments, Tujunga, CA, USA). A stainless steel needle (0.28 mm o.d.) was inserted unilaterally through a small hole in the skull, and the needle tip was placed in the left medial forebrain bundle (MFB). The stereotaxic coordinates according to the atlas of Paxinos and Watson [49] were as follows: anterior–posterior (A/P) = −2.8 mm, lateral (L) = +1.8 mm, and dorsal–ventral (D/V) = −8.6 mm. 6-OHDA at a dose of 16 μg (calculated as the free base) was dissolved in a volume of 4 μL of sterile 0.9% NaCl supplemented with 0.05% ascorbic acid. The solution was freshly prepared before surgery. Then, it was slowly infused into the left MFB at a flow rate of 0.5 μL/min using a 10 μL Hamilton syringe. After stopping the infusion of 6-OHDA, the cannula was left in place for an additional 5 min to allow complete diffusion of the toxin, and then, it was slowly retracted. Control rats were treated in the same manner, but received equivalent volumes of the vehicle instead of 6-OHDA.

### 2.5. Sucrose Preference Test

The sucrose preference test (SPT) is commonly used as a measure of anhedonia in rodents [50,51]. Rats were transferred individually to single housing cages with free access to food and tap water. During a 24 h training phase, each rat was provided with two pre-weighed bottles of water. After the training day, one of the bottles was switched to the one containing 3% sucrose solution, and after 24 h, the bottles’ positions were reversed to avoid position-specific effects. The bottles were weighed and filled with fresh liquids daily. In the SPT, 58 rats were used (10 sham-operated and 48 6-OHDA-lesioned animals). The percentage sucrose consumption was calculated according to the formula (% sucrose preference = sucrose intake × 100/total intake). The sum of water and sucrose consumption was defined as total liquid intake. During the 3-day sucrose preference test, intake of water or sucrose solution was recorded daily between 9:00 and 11:00 a.m. After termination of the sucrose preference test, each rat was transferred from a single breeding cage to a common home cage with free access to drinking water and standard laboratory chow. The sucrose preference test was performed 4 times during the entire experimental procedure (see Figure 1). The analysis of the percentage of sucrose solution consumption by rats from the studied groups was performed only in the rats that were verified based on the rotation test and on the analysis of tissue concentrations of dopamine (DA) in the striatum and substantia nigra described previously [43].

### 2.6. Rotational Behavior

A detailed description of the measurement of rotational behavior in the groups of rats studied in the present work and the results of analysis of these behaviors after acute and chronic administration of AMI and L-DOPA, alone or in combination, were presented in our previous report [43].

### 2.7. Determination of Monoamine Levels in the Brain Tissue Homogenates

The tissue concentrations of noradrenaline (NA), DA and its metabolites, 3,4-dihydroxyphenylacetic acid (DOPAC), 3-methoxytyramine (3-MT) and homovanillic acid (HVA), as well as 5-HT and its metabolite 5-hydroxyindoleacetic acid (5-HIAA), were assayed in tissue homogenates of PFC and HIP, separately for the left (lesioned) and right (intact) sides, by reverse-phase high-performance liquid chromatography (HPLC) with coulometric detection. To determine monoamine levels, 48 unilaterally 6-OHDA-lesioned rats were used. Briefly, tissue samples were weighed and homogenized in ice-cold 0.1 M perchloric acid containing 0.05 mM ascorbic acid. After centrifugation (10,000× *g*, 10 min), the supernatants were filtered through 0.2 μm cellulose filters (Alltech Associates Inc., Deerfield, IL, USA) and injected into the HPLC system, which consisted of a P680 pump, an ASI-100 autosampler, and a thermostated column compartment TCC-100 (Dionex, Germering, Germany) equipped with a Hypersil C18 column (100 × 3 mm i.d., 3 µm particle size) fitted with a 10 × 3 mm precolumn (Thermo Fisher Scientific Inc., Waltham, MA, USA). Detection was conducted by use of a Coulochem III detector (ESA Inc., Chelmsford, MA, USA) equipped with a guard cell (ESA 5020, electrode potential set at 600 mV) and a dual electrode analytical cell (ESA 5010, applied potential E1 = −200 mV, E2 = 300 mV). The mobile phase consisted of 35 mM citrate/47 mM disodium phosphate buffer (pH 4.2), supplemented with 0.25 mM EDTA, 0.25 mM 1-octanesulfonic acid sodium salt, 2.4% methanol, and 1.3% acetonitrile. The temperature of the analytic cell and the column was maintained at 30 °C and the flow rate was maintained at 0.8 mL/min. All neurotransmitters and metabolites were quantified by peak area comparisons of the tested samples with freshly prepared standards, run on the day of analysis. The approximate retention times of the measured substances were as follows: for NA = 2.31, DOPAC = 4.26, DA = 5.31, 5-HIAA = 7.98, HVA = 12.53, 5-HT = 14.2, and 3-MT = 15.05 min. Data were collected and analyzed using Chromeleon 6.8 software (Dionex, Germering, Germany).

### 2.8. Autoradiography—Tissue Preparation

Coronal sections (10 µm) of the PFC, HIP and NAcc (core and shell) were cut using a cryostat microtome at −20 °C. The sections were then thaw mounted on gelatin-coated microscopic slides and stored at −20 °C until use. For the autoradiographic study, 40 rats were used (8 sham-operated and 32 6-OHDA-lesioned animals). In cases of mechanical damage to brain cross-sections, they were excluded from the autoradiographic analysis.

#### 2.8.1. The Binding of [^3^H]GBR 12,935 to DAT

The binding of [^3^H]1-[2-(diphenyl-methoxy)-ethyl]-4-(3-phenylpropyl)piperazine ([^3^H]GBR 12,935) to DAT was assayed as previously described [43,52,53]. Thawed and dried tissue sections were incubated at 4 °C for 20 h in 50 mM Tris-HCl buffer (pH = 7.5), containing 300 mM NaCl, 0.2% bovine serum albumin, 1 µM cis-flupentixol, and 2 nM [^3^H]GBR 12,935 (Perkin Elmer Life and Analytical Sciences, Boston, MA, USA, 40 Ci/mmol). The non-specific binding was assessed on consecutive sections in the presence of 20 µM mazindol. The sections were rapidly rinsed 5 times for 30 s with 50 mM Tris-HCl buffer (pH = 7.5) containing 450 mM NaCl at 4 °C. The tissue sections were immersed once in ice-cold deionized water, dried, and then exposed together with radioactive standards ([^3^H] microscales, ARC, USA) to Kodak BioMax MR film for 4 weeks at 4 °C.

#### 2.8.2. The Binding of [^3^H]citalopram to SERT

The binding of [^3^H]citalopram to SERT was assayed as previously described [43,53,54,55]. The tissue sections were incubated for 30 min at 4 °C in 50 mM Tris-HCl buffer (pH = 7.4), containing 120 mM NaCl, 5 mM KCl and 2 nM [^3^H]citalopram (Perkin Elmer Life and Analytical Sciences, Boston, MA, USA, 84.5 Ci/mmol). The non-specific binding was assessed in the presence of 20 µM fluoxetine. The sections were rapidly rinsed two times for 2 min with 50 mM Tris-HCl buffer (pH = 7.5) at 4 °C. The tissue sections were once immersed in ice-cold deionized water, dried, and then exposed together with radioactive standards ([^3^H] microscales, ARC, USA) to Kodak BioMax MR film for 4 weeks at 4 °C.

#### 2.8.3. The Binding of [^3^H]nisoxetine to NET

The binding of [^3^H]nisoxetine to NET was assayed as previously described [43,54]. Thawed and dried tissue sections were preincubated for 15 min at 23–25 °C in 50 mM Tris-HCl buffer (pH = 7.4), containing 300 mM NaCl, 5 mM KCl. Afterwards, the tissue sections were incubated for 2 h in ice-cold pre-incubation buffer, containing 3 nM [^3^H]nisoxetine (Perkin Elmer Life and Analytical Sciences, Boston, MA, USA, 82 Ci/mmol). The non-specific binding was assessed in the presence of 10 µM mazindol. The sections were rapidly rinsed 3 times for 2 min with ice-cold pre-incubation buffer. The tissue sections were immersed once in cold deionized water, dried, and then exposed together with radioactive standards ([^3^H] microscales, ARC, USA) to Kodak BioMax MR film for 10 weeks at 4 °C.

#### 2.8.4. Autoradiographic Analysis of the Radioligands Binding to DAT, SERT, and NET

Films were developed with a Dectol developer (Kodak, Sigma-Aldrich, St. Louis, MO, USA), fixed with a GBX fixer/replenisher (Kodak, Sigma-Aldrich, St. Louis, MO, USA), and dried. Autoradiograms were scanned and analyzed by a computer program, Multi Gauge 3.0 (Fujifilm Europe, GmbH, Warsaw, Poland). To quantify the ligand binding density, the optical density of the co-exposed radioactive standards ([^3^H]microscales, ARC, USA) was determined. The use of standards and the derived standard curve allowed for the conversion of the areal optical density into nCi of the radioligand bound/mg tissue. The specific binding of [^3^H]GBR 12,935, [^3^H]citalopram and [^3^H]nisoxetine, after subtraction of non-specific binding, was estimated in the NAcc at levels from the AP = 1.44 to 1.92 mm, in the HIP (CA1, CA3, DG) at levels from the AP = −2.28 to −3.48 mm, in the PFC at levels from the AP = 3.72 to 2.52 mm from the bregma, according to the stereotaxic atlas of Paxinos and Watson [48]. The binding of labeled ligands at the above-mentioned NAcc, HIP, and PFC levels was examined separately for the lesioned and intact sides.

### 2.9. Statistical Analysis

The statistical analysis of the obtained behavioral data (sucrose preference test) was performed using the repeated measures ANOVA, followed (if significant) by the Duncan test for post hoc comparisons. Neurochemical parameters, such as monoamine levels and radioligand binding to their transporters, were analyzed in the unilaterally 6-OHDA-lesioned groups of rats treated chronically with AMI and L-DOPA using a two-way ANOVA followed by the Newman–Keuls post hoc test only if a significant interaction between the tested drugs was found. The rates of monoamine oxidase (MAO)-dependent and catechol-O-methyltransferase (COMT)-dependent catabolic pathways as well as the total DA catabolism were evaluated, as the concentration ratios of intracellular metabolite DOPAC to DA, extracellular metabolite 3-MT to DA, and the final DA metabolite HVA to DA were expressed as the catabolism rate indices of (DOPAC/DA) × 100, (3-MT/DA) × 100 and (HVA/DA) × 100, respectively. The total rate of 5-HT catabolism was calculated from the ratio of its metabolite 5-HIAA to 5-HT concentration and was expressed as the catabolism rate index (5-HIAA/5-HT) × 100. The indices were calculated using concentrations from individual tissue samples.

The significance of differences between the lesioned (left) and intact (right) sides within each examined group of the PFC and HIP was estimated by Student’s *t*-test for dependent samples. Moreover, the significance of differences in the radioligand binding to their transporters between the sham-operated group and the 6-OHDA-lesioned control group on the lesioned or intact sides was assessed by Student’s *t*-test for independent samples.

The *p*-values of less than or equal to 0.05 were considered to indicate statistical significance. The statistical analysis was performed using STATISTICA 10.0 software (Statsoft, Inc., Tulsa, OK, USA).

## 3. Results

### 3.1. Consumption of 3% Sucrose Solution in Unilaterally 6-OHDA-Lesioned Rats Treated Chronically with AMI and L-DOPA, Alone or in Combination

**Figure 3:**
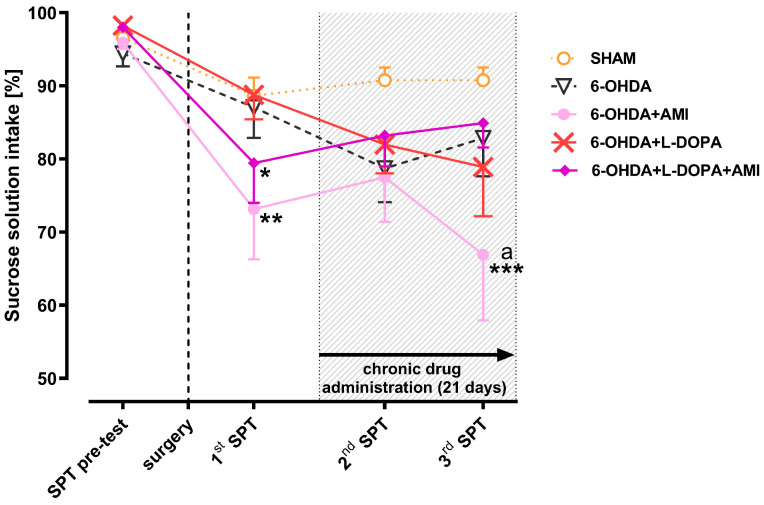
The effect of repeated administrations of AMI (10 mg/kg i.p.) and L-DOPA (12 mg/kg i.p.), separately or in combination, on sucrose preference in rats receiving a single unilateral injection of 6-OHDA at a dose of 16 μg/4 μL into the MFB without desipramine shielding. Control rats received solvent instead of 6-OHDA into the MFB. The sham-operated group and 6-OHDA control received i.p. vehicle. Results are presented as means ± SEM; n—number of rats per group: n = 10 for sham+veh-; n = 10 for 6-OHDA+veh-; n = 9 for 6-OHDA+AMI-; n = 14 for 6-OHDA+L-DOPA-; n = 15 for the 6-OHDA+AMI+L-DOPA-treated group. The statistical analysis of the obtained data was performed using repeated-measures ANOVA, followed by Duncan’s post hoc test: * *p* < 0.05, ** *p* < 0.01, *** *p* < 0.001 vs. pretest, ^a^ *p* < 0.05 vs. control group after test 3.

The preference for drinking 3% sucrose solution was measured four times: 1 week before surgery (pretest), then after 1 (test 1), 3 (test 2), and 4.5 weeks (test 3) after the unilateral administration of 6-OHDA into the MFB (Figure 3).

Sucrose intake preference tests 2 and 3 were conducted while the tested drugs, AMI and L-DOPA, were administered chronically, either separately or in combination, for 21 days (Figure 1 and Figure 3). Chronic treatment with these drugs began 2 weeks after administration of 6-OHDA. L-DOPA has a short half-life, resulting in significant fluctuations in its plasma concentrations [56]. AMI, on the other hand, has a longer half-life; therefore, elevated plasma concentrations of this drug persist for a much longer period, which is also influenced by its metabolism in the liver [57]. Therefore, the 24 h measurement of sucrose solution intake during test 2 or 3, presented in Figure 3, reflects the effects of L-DOPA alone or the combination of AMI+L-DOPA in the relatively short “on” phase, i.e., during the action on motor functions (occurrence of rotation) [43], as well as in the much longer “off” phase, i.e., in the absence of rotation behavior but at the prolonged action of AMI.

As shown in Figure 3, in the first week after administration of 6-OHDA into the MFB (test 1), only two groups of rats (6-OHDA+AMI; 6-OHDA+AMI+L-DOPA) showed a significant decrease in intake of the 3% sucrose solution compared to the pretest intake level. After 7 days of treatment with the test drugs (test 2), in the first group receiving AMI alone and in the second group receiving AMI in combination with L-DOPA, the consumption of 3% sucrose solution improved, remaining at a similar level, although slightly lower than in the sham-operated control group receiving i.p. vehicle. Also, in the group administered L-DOPA alone, at that time point, intake of a 3% sucrose solution was within the same range. However, sucrose intake assessed in test 3 in the group receiving AMI alone, i.e., after 2.5 weeks of repeated administration of this medication, significantly decreased compared to the intake in the sham-operated control group at the same time point (Figure 3). This decrease in sucrose intake in the AMI-treated group was also significantly lower than that measured in the pretest (Figure 3). In contrast to the group receiving AMI alone, in the AMI+L-DOPA-treated group, 3% sucrose solution intake measured in test 3 increased. In fact, at this time point, its level was close to that in the sham-operated control group and slightly higher than in the 6-OHDA-lesioned group repeatedly treated with L-DOPA alone (Figure 3).

### 3.2. The Effects of Chronic Treatment with AMI and L-DOPA, Alone or in Combination, on Concentrations of DA, 5-HT, and NA in the HIP and PFC of Unilaterally 6-OHDA-Lesioned Rats

#### 3.2.1. The Concentrations of DA and Its Metabolites in the HIP and PFC

**Figure 4:**
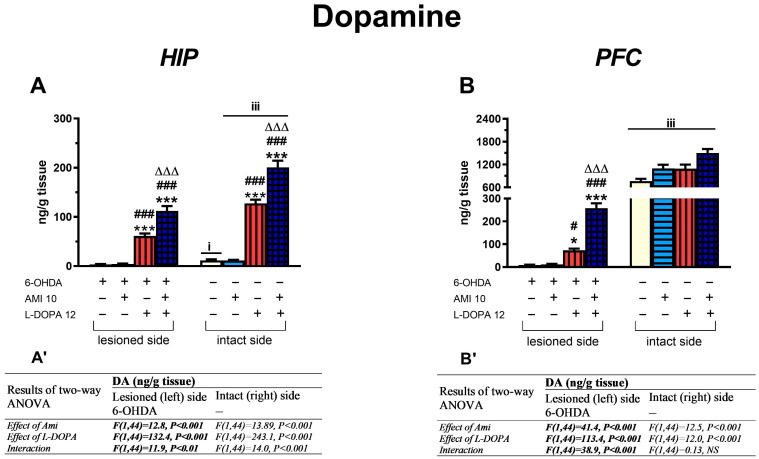
Concentrations of dopamine (DA) in the lesioned (**left**) and intact (**right**) HIP and PFC of rats receiving a single unilateral injection of 6-OHDA at a dose of 16 μg/4 μL into the MFB without desipramine shielding, determined 1 h after the last chronic doses of AMI (10 mg/kg i.p.) and L-DOPA (12 mg/kg i.p.). The data are presented as the mean ± SEM; n—number of rats per group: n = 10 for 6-OHDA+veh-; n = 9 for 6-OHDA+AMI-; n = 14 for 6-OHDA+L-DOPA-; n = 15 for the 6-OHDA+AMI+L-DOPA-treated group. Statistical analysis of the data presented in Figures (**A**,**B**) was performed using a two-way ANOVA, and its effects are included below in Tables (**A’**,**B’**). The abbreviation of NS denotes a nonsignificant effect. Symbols indicate the significance of differences according to the Newman–Keuls post hoc test: * *p* < 0.05, *** *p* < 0.001 vs. the 6-OHDA+veh-; ^#^ *p* < 0.05, ^###^ *p* < 0.001 vs. the 6-OHDA+AMI-; ^ΔΔΔ^ *p* < 0.001 vs. the 6-OHDA+L-DOPA-treated group, within the corresponding lesioned or intact sides. These symbols were placed above appropriate bars only when a significant interaction occurred. Letters indicate statistically significant differences estimated by Student’s *t*-test for dependent samples: ^iii^ *p* < 0.001 vs. the corresponding group on the lesioned side. ^i^ vs. the corresponding group on the lesioned side (i.e., 6-OHDA + veh; 6-OHDA + AMI; 6-OHDA + L-DOPA; 6-OHDA + AMI + L-DOPA).

The results of a two-way ANOVA performed for DA level in the lesioned (left) and intact (right) HIP, and in the lesioned PFC, revealed significant treatment effects of AMI and L-DOPA alone, as well as an interaction of AMI × L-DOPA, as presented in Figure 4. However, in the intact PFC, although there were significant treatment effects of AMI and L-DOPA, there was no significant interaction between the tested drugs (Figure 4B,B’).

In general, post hoc comparisons of DA levels between the examined groups in the lesioned and intact HIP, as well as in the lesioned PFC, showed that chronic treatment with L-DOPA alone or with AMI+L-DOPA combination, significantly increased tissue DA concentration compared to corresponding groups treated with vehicle or AMI alone (Figure 4A,B). Furthermore, joint administration of AMI and L-DOPA increased the tissue DA concentration on both sides of the HIP and in the lesioned PFC more distinctly than did L-DOPA alone (Figure 4A,B). In the intact PFC, although no significant interaction between the studied drugs was found, treatment effects showed that both L-DOPA and AMI given alone increased DA content equally, but the effect was most pronounced when these drugs were administered in combination.

Concomitantly with changes in DA content, chronic treatment with AMI and L-DOPA significantly affected the tissue concentration of DA metabolites in the PFC and HIP (Table 1). The F values for the two-way ANOVA performed for the concentrations of DOPAC, 3-MT, and HVA, as well as for the catabolic indexes, such as the DOPAC-to-DA (DOPAC/DA), 3-MT-to-DA (3-MT/DA), and HVA-to-DA (HVA/DA) concentration ratios, illustrating the rate of the MAO-dependent oxidative DA catabolism, COMT-dependent DA catabolism, and the total DA catabolism on the lesioned and intact side of PFC or HIP are presented in Table 1 and Table 2, respectively.

Regarding the tissue concentrations of DA metabolites, post hoc analysis showed that chronic treatment with L-DOPA alone or in combination with AMI increased tissue concentrations of DOPAC and HVA in the lesioned and intact HIP, as well as in the lesioned PFC, compared to groups treated i.p. with vehicle or AMI alone (Table 1). Moreover, in these brain structures, the increases in DOPAC and HVA tissue content after combined administration of the studied drugs were significantly higher than those observed after administration of L-DOPA alone (Table 1). In the intact PFC, a two-way ANOVA revealed only significant treatment effects of AMI and L-DOPA alone on DOPAC and HVA levels. Both drugs increased the tissue concentrations of these DA metabolites in the intact PFC. As to the tissue contents of 3-MT in the lesioned and intact PFC and HIP, it was affected only by L-DOPA treatment, which significantly increased its level in both these brain structures.

In general, chronic treatment with L-DOPA alone or in combination with AMI accelerated both oxidative and total DA catabolism assessed by DOPAC/DA and HVA/DA concentration ratios, both in the lesioned and intact PFC and HIP (Table 2). In contrast, COMT-dependent DA catabolism, assessed by 3-MT/DA, was attenuated by AMI and L-DOPA mainly in the lesioned and intact HIP (Table 2).

In the lesioned PFC, a two-way ANOVA for the DOPAC/DA metabolic index revealed significant treatment effects of AMI and L-DOPA alone, with no interaction between these drugs. In fact, on this side of the PFC, AMI decreased the DOPAC/DA index, while L-DOPA increased it. As to the 3-MT/DA metabolic index in the lesioned PFC, a two-way ANOVA revealed treatment effects neither with AMI and L-DOPA nor an interaction between these drugs. In turn, a two-way ANOVA for HVA/DA metabolic index in the lesioned PFC showed significant treatment effects of AMI and L-DOPA, as well as an interaction between these drugs. Consistently, on this side of PFC, the HVA/DA index increased in the L-DOPA-treated group compared with the groups treated i.p. with vehicle or AMI alone. However, in the group receiving AMI+L-DOPA, a significant decrease in the HVA/DA metabolic index was observed compared to the group treated with L-DOPA alone.

In the intact PFC, a two-way ANOVA for the DOPA/DA and HVA/DA metabolic indices revealed only a significant treatment effect of L-DOPA. This drug increased these indices in groups receiving L-DOPA alone or in combination with AMI (Table 2). Regarding the 3-MT/DA metabolic index in the intact PFC, a two-way ANOVA revealed significant treatment effects of AMI and L-DOPA, but no interaction between these drugs.

In the lesioned and intact HIP, a two-way ANOVA performed for DOPAC/DA and 3-MT/DA metabolic indices revealed significant treatment effects of AMI and L-DOPA, as well as an interaction between these drugs. Post hoc comparison showed that AMI decreased the DOPAC/DA metabolic index compared to the vehicle-treated group. However, L-DOPA alone and the combination AMI+L-DOPA increased DOPAC/DA metabolic index compared to the AMI-treated group. In contrast, the metabolic index 3-MT/DA in the lesioned HIP decreased in comparison with the groups treated with vehicle or AMI alone (Table 2). In the intact HIP, this index decreased only compared to the group receiving the vehicle.

A two-way ANOVA for the HVA/DA metabolic index in the lesioned and intact HIP showed only a significant treatment effect of L-DOPA; this drug increased the HVA/DA index in the groups receiving L-DOPA alone or in combination with AMI (Table 2).

#### 3.2.2. The Concentrations of 5-HT and Its Metabolite 5-HIIA in the PFC and HIP

**Figure 5:**
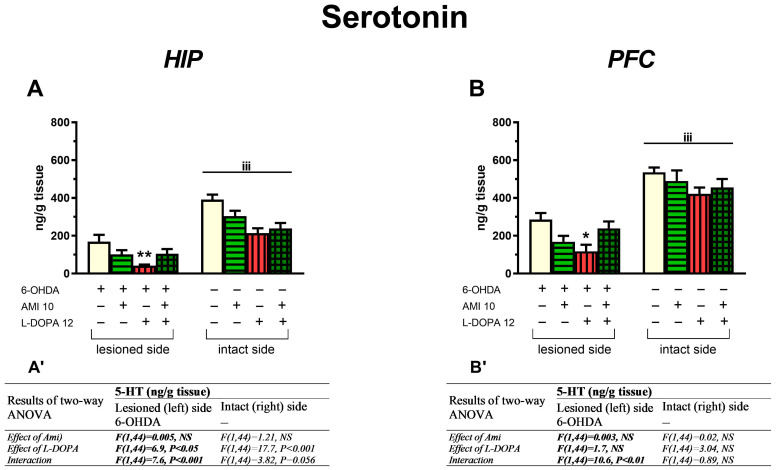
Concentrations of serotonin (5-HT) in the lesioned and intact hippocampus (HIP) and prefrontal cortex (PFC) of rats receiving a single unilateral injection of 6-OHDA at a dose of 16 μg/4 μL into the MFB without desipramine shielding, determined 1 h after the last chronic doses of AMI (10 mg/kg i.p.) and L-DOPA (12 mg/kg i.p.). The data are presented as the mean ± SEM; n—number of rats per group: n = 10 for 6-OHDA + veh-; n = 9 for 6-OHDA + AMI-; n = 14 for 6-OHDA + L-DOPA-; n = 15 for the 6-OHDA + AMI + L-DOPA-treated group. Statistical analysis of the data presented in Figures (**A**,**B**) was performed using a two-way ANOVA, and its effects are included below in Tables (**A’**,**B’**). The abbreviation of NS denotes a nonsignificant effect. Symbols indicate the significance of differences according to the Newman–Keuls post hoc test when a significant interaction occurred: * *p* < 0.05, ** *p* < 0.01 vs. 6-OHDA + veh-treated group. Letters indicate statistically significant differences estimated by Student’s *t*-test for dependent samples: ^iii^ *p* < 0.001 vs. the corresponding group on the lesioned side.

In the lesioned HIP, a two-way ANOVA performed for 5-HT level demonstrated a significant treatment effect of L-DOPA, and no treatment effect of AMI, but a pronounced interaction between these two drugs (Figure 5A,A’). The post hoc analysis showed that L-DOPA alone decreased 5-HT content, but its combined administration with AMI reversed this effect (Figure 5A). In the intact HIP, this analysis showed only a significant treatment effect of L-DOPA; this drug decreased 5-HT content in groups receiving L-DOPA alone and the combination of AMI+L-DOPA (Figure 5A,A’).

A two-way ANOVA for 5-HT concentration in the lesioned PFC revealed a significant interaction between AMI and L-DOPA, but no treatment effects of these drugs when administered alone (Figure 5B). Post hoc comparison of the examined groups showed that L-DOPA alone significantly decreased 5-HT content in the lesioned PFC while its coadministration with AMI increased it, reaching almost the same level as that observed in the 6-OHDA-lesioned group treated with vehicle (Figure 5B). In the intact PFC, a two-way ANOVA revealed neither the treatment effect of AMI and L-DOPA nor the interaction between the tested drugs.

In the lesioned PFC, two-way ANOVA of 5-HIAA concentration showed no treatment effects of AMI and L-DOPA administered alone, and a significant interaction (Table 3). Post hoc comparison showed that in the group receiving AMI+L-DOPA, the concentration of 5-HIAA was significantly higher than in the groups receiving vehicle, AMI, or L-DOPA alone.

In the lesioned PFC, a two-way ANOVA for MAO-dependent 5-HT catabolism assessed using the 5-HIAA/5-HT metabolic index revealed a lack of treatment effect of AMI, a significant effect of L-DOPA, and an interaction between AMI and L-DOPA. Post hoc comparison showed that L-DOPA alone increased the 5-HIAA/5-HT metabolic index compared to groups receiving vehicle or AMI. However, the combined administration of AMI+L-DOPA decreased this metabolic index compared to the L-DOPA-treated group (Table 3).

In the intact PFC, a two-way ANOVA revealed only the treatment effect of L-DOPA on both the 5-HIAA concentration and on the 5-HIAA/5-HT metabolic index. In groups receiving L-DOPA alone or AMI+L-DOPA, both the 5-HIAA concentration and the metabolic index 5-HIAA/5-HT were enhanced.

In the lesioned and intact HIP, a two-way ANOVA for 5-HIAA concentration revealed no treatment effects of AMI and L-DOPA, and a significant interaction (Table 3). A post hoc comparison showed that L-DOPA alone decreased the 5-HIAA level in the lesioned HIP compared to the vehicle-treated group while the combination of AMI+L-DOPA increased it compared to the L-DOPA-treated group.

A two-way ANOVA for 5-HIAA/5-HT metabolic index in the lesioned and intact HIP showed only a treatment effect of L-DOPA. This metabolic index increased in groups receiving L-DOPA alone or AMI+L-DOPA (Table 3).

#### 3.2.3. The Concentration of NA in the HIP and PFC

**Figure 6:**
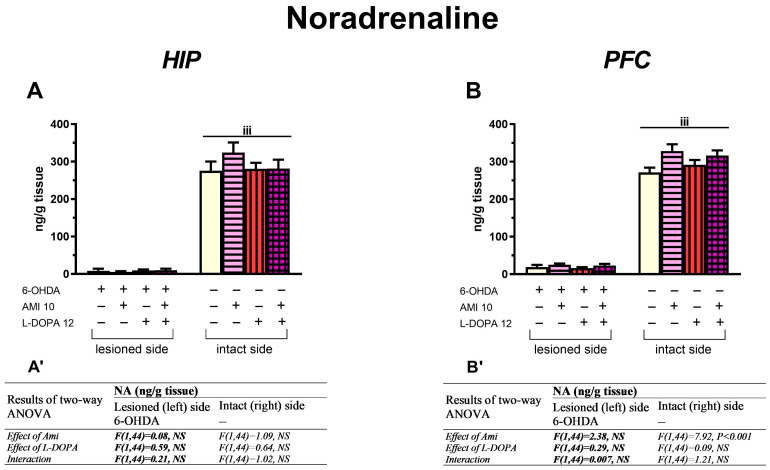
Concentrations of noradrenaline (NA) in the lesioned and intact hippocampus (HIP) and prefrontal cortex (PFC) of rats receiving a single unilateral injection of 6-OHDA at a dose of 16 μg/4 μL into the MFB without desipramine shielding, determined 1 h after the last chronic doses of AMI (10 mg/kg i.p.) and L-DOPA (12 mg/kg i.p.). Effects of this analysis are included in Tables (**A’**,**B’**). The abbreviation of NS denotes a nonsignificant effect. Letters indicate statistically significant differences estimated by Student’s *t*-test for dependent samples: ^iii^ *p* < 0.001 vs. the corresponding group on the lesioned side.

In the lesioned and intact HIP, as well as in the lesioned PFC, a two-way ANOVA performed for NA concentration revealed a lack of significant treatment effects of AMI and L-DOPA, and no interaction between the tested drugs (Figure 6A,A’). Neither AMI (10 mg/kg) nor L-DOPA (12 mg/kg), administered alone or in combination, affected the tissue concentration of NA in both sides of HIP and in the lesioned PFC (Figure 6A,B).

However, in the intact PFC, this analysis demonstrated only a significant treatment effect of AMI; this drug increased the NA content in groups receiving AMI alone or the AMI+L-DOPA combination (Figure 6B,B’).

#### 3.2.4. The Comparative Analysis of Monoamine Concentrations in the PFC and HIP of 6-OHDA-Lesioned Rats Treated with AMI and L-DOPA

The effects of a unilateral lesion to the noradrenergic, dopaminergic, and serotonergic systems induced by 6-OHDA injection into the left MFB, as well as of chronic administration of AMI and L-DOPA, alone or in combination, on monoamine concentrations in the PFC and HIP are summarized in Table 4.

In our previous study, it was demonstrated that 6-OHDA administration at a dose of 16 µg/4 µL unilaterally into the MFB, without desipramine shielding, caused drastic decreases in the concentrations of NA and DA as well as moderate decreases in 5-HT concentration (by more than 50%) in the lesioned left HIP and PFC compared to the left HIP and PFC of the sham-operated control rats [40]. In the intact right sides of the HIP and PFC, DA and 5-HT concentrations remained at similar control levels in both examined groups [40]. Interestingly, the administration of 6-OHDA into the left MFB reduced NA content in the intact right HIP and PFC (by 25% and 15%, respectively) compared to the NA concentration in the intact right side of these brain structures in the sham-operated group [40].

In light of the above data, it can be assumed that in this study, the concentrations of DA and 5-HT on the intact right side may be adequate control for the changes in the concentrations of these neurotransmitters on the lesioned left side induced by 6-OHDA. However, the concentrations of NA on the intact side of the HIP and PFC only partially meet the criterion for adequate control because 6-OHDA administration into the left MFB caused some small decreases in the contents of this neurotransmitter in the studied brain structures on the intact right side. Therefore, to display all 6-OHDA-induced changes in monoamine levels, Table 4 reports decreases in NA content on the intact side of PFC and HIP, based on our earlier study [40].

In the present study, chronic administration of AMI and L-DOPA, alone or in combination, coincided with the period of pathological alterations in the functioning of monoaminergic systems in the examined brain structures, caused by a unilateral 6-OHDA administration into the left MFB. The administration of these drugs did not alter the drastic decline of NA concentration on the lesioned side of the PFC and HIP. In contrast, in the intact PFC, chronic administration of AMI alone or in combination with L-DOPA increased NA concentrations; however, no such effect was observed in the intact HIP.

Chronic treatment with AMI did not affect the 6-OHDA-induced decrease in DA concentration in the lesioned PFC and HIP. In the intact PFC, DA content in the group receiving AMI alone was increased; its level was almost the same as in the group treated with L-DOPA alone. In general, in the intact HIP, DA concentration was very low, and AMI alone did not change it. However, chronic treatment with L-DOPA alone or with the AMI+L-DOPA combination increased DA concentrations in the lesioned and intact PFC and HIP. Furthermore, in the lesioned PFC and in the lesioned and intact HIP, a significant interaction between AMI and L-DOPA was found increasing DA content more significantly than L-DOPA alone.

In the lesioned PFC and HIP, chronic administration of AMI did not significantly alter the moderate decrease in 5-HT concentration evoked by 6-OHDA. AMI also did not change 5-HT concentration in the intact PFC and HIP. However, in the lesioned PFC and HIP, chronic treatment with L-DOPA alone decreased 5-HT content, whereas chronic combined administration of AMI with L-DOPA increased it, due to drug interactions. In the intact PFC, neither AMI, L-DOPA, nor AMI+L-DOPA affected the control level of 5-HT. However, in the intact HIP, both L-DOPA alone and the AMI+L-DOPA combination decreased 5-HT concentration.

### 3.3. The Binding of the Radioligands to DAT, SERT, and NET in the Nucleus Accumbens

**Figure 7:**
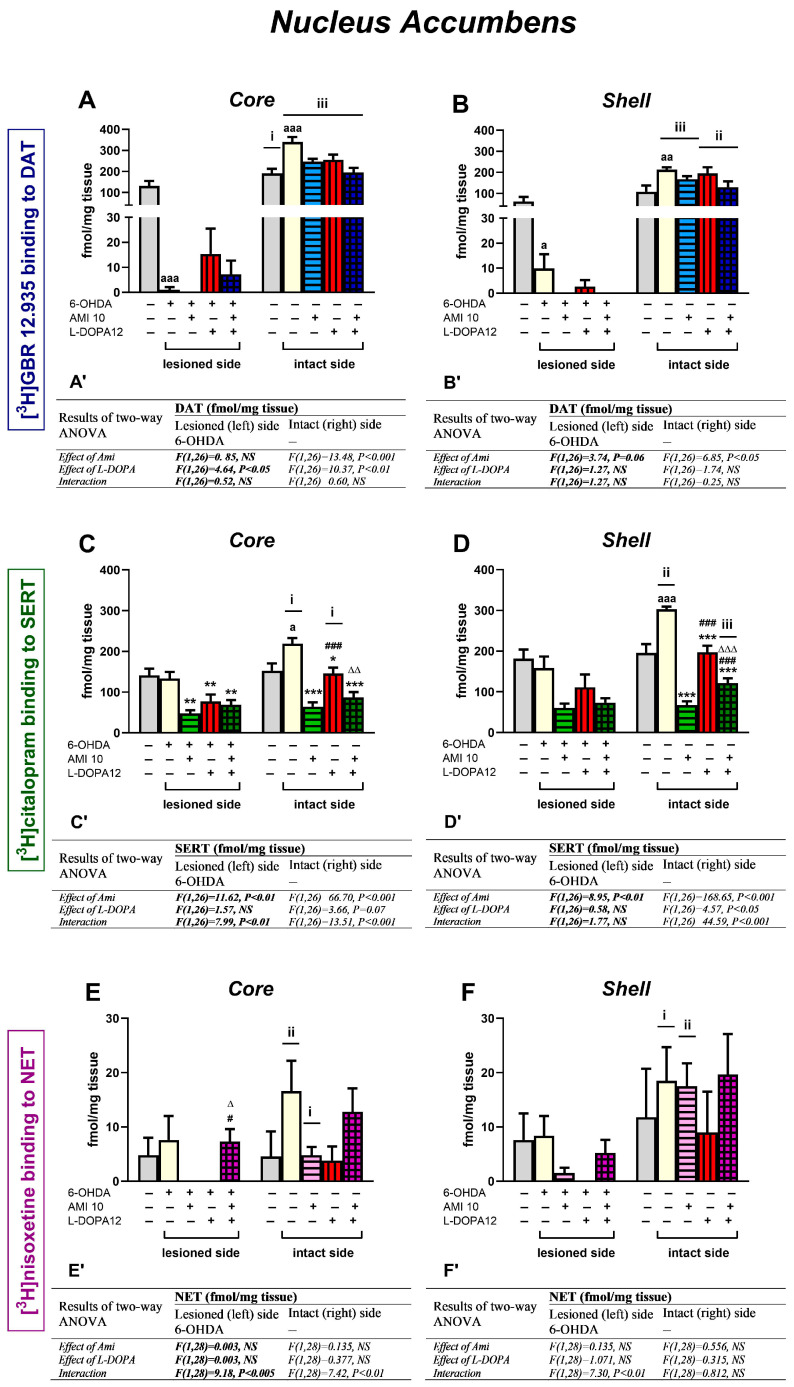
The effects of chronic (21 days) administration of AMI (10 mg/kg i.p.) and L-DOPA (12 mg/kg i.p.), alone and in combination, on the binding of [^3^H]GBR 12,935 to DAT (**A**,**B**), [^3^H] citalopram to SERT (**C**,**D**), and [^3^H] nisoxetine to NET (**E**,**F**) in the nucleus accumbens core and shell. Data are presented as the mean ± SEM; n = 6–8 rats per group. Statistical analysis of the radioligand binding to monoamine transporters was performed using a two-way ANOVA, separately for the lesioned and intact sides of the core and shell in four groups of rats receiving 6-OHDA unilaterally into the left MFB and the tested drugs i.p. The F values for these analyses are presented in Tables (**A’**–**F’**) below Figure 7A–F. Symbols indicate significance of differences according to the Newman–Keuls post hoc test: * *p* < 0.05, ** *p* < 0.01, *** *p* < 0.001 vs. 6-OHDA+ veh-; ^#^ *p* < 0.05, ^###^ *p* < 0.001 vs. 6-OHDA+AMI-; ^Δ^ *p* < 0.05, ^ΔΔ^ *p* < 0.01, ^ΔΔΔ^ *p* < 0.001 vs. 6-OHDA+L-DOPA-treated group. The letter “i” indicates statistically significant differences estimated by Student’s *t*-test for dependent samples: ^i^ *p* < 0.05, ^ii^ *p* < 0.01, ^iii^ *p* < 0.001 vs. the corresponding group on the lesioned side. The letter “a” indicates statistically significant differences estimated by Student’s *t*-test for independent samples: ^a^ *p* < 0.05, ^aa^ *p* < 0.01, ^aaa^ *p* < 0.001 vs. the sham-operated group.

Quantitative analysis of the binding of [^3^H]GBR 12,935 to DAT, [^3^H]citalopram to SERT, and [^3^H]nisoxetine to NET in unilaterally 6-OHDA-lesioned groups of rats was performed in coronal sections of the core and shell of nucleus accumbes (NAcc), 1 h after administration of the last doses of AMI and L-DOPA, alone or in combination (Figure 7A–F).

#### 3.3.1. The Binding of [^3^H]GBR 12,935 to DAT

Unilateral administration of 6-OHDA into the left MFB drastically decreased [^3^H]GBR 12,935 binding to DAT in the left core and shell of NAcc compared to the corresponding NAcc subregions in the sham-operated group (for left core, t = 5.67, df = 14, *p* < 0.001; for left shell, t = 2.274, df = 14, *p* < 0.05). Moreover, such administration of 6-OHDA evoked up-regulation of [^3^H]GBR 12,935 binding to DAT in the right core and shell of the NAcc compared to the corresponding NAcc subregions in the sham-operated group (for right core, t = −4.05, df = 14, *p* < 0.001; for right shell, t = −3.243, df = 14, *p* < 0.01) (Figure 7A,B).

A two-way ANOVA for [^3^H]GBR 12,935 binding to DAT performed in four groups of 6-OHDA-lesioned rats (6-OHDA+veh-, 6-OHDA+AMI-, 6-OHDA+L-DOPA-, and 6-OHDA+AMI+L-DOPA) in the left core revealed only the treatment effect of L-DOPA; this drug slightly increased this binding (Figure 7A). The same analysis performed for [^3^H]GBR 12,935 binding to DAT in the intact core showed treatment effects of AMI and L-DOPA, but no interaction between these drugs (Figure 7A). On this side of the NAcc core, both these drugs decreased DAT binding. In the intact NAcc shell, a two-way ANOVA showed only a treatment effect of AMI, namely, the decreased DAT binding was observed in groups receiving chronic i.p. injections of AMI alone or the AMI+L-DOPA combination (Figure 7B).

#### 3.3.2. The Binding of [^3^H]citalopram to SERT

Unilateral administration of 6-OHDA into the left MFB did not affect [^3^H]citalopram binding to SERT in the left core and shell of NAcc compared to the corresponding NAcc subregions in the sham-operated group (Figure 7C,D). However, such administration of 6-OHDA evoked up-regulation of [^3^H]citalopram binding to SERT in the right core and shell (Figure 7C,D) compared to the corresponding NAcc subregions in the sham-operated group (for right core, t = −2.862, df = 13, *p* < 0.05; for right shell, t = −4.302, df = 12, *p* < 0.001).

A two-way ANOVA performed for the [^3^H]citalopram binding to SERT in the left and right core revealed a treatment effect of AMI, a lack of treatment effect of L-DOPA, and a significant interaction between the tested drugs (Figure 7C). The same analysis for the radioligand binding to SERT in the lesioned shell showed only the treatment effect of AMI, whereas on the intact side, both significant treatment effects of AMI and L-DOPA, as well as an interaction, was found (Figure 7D). Post hoc comparisons performed on both sides of the core and on the intact side of the shell showed that AMI administration alone or in combination with L-DOPA equally reduced [^3^H]citalopram binding to SERT compared to the 6-OHDA-lesioned group receiving i.p. vehicle. L-DOPA administration alone also significantly reduced [^3^H]citalopram binding in the above-mentioned NAcc subregions; however, on the intact side, these decreases were much smaller compared to the 6-OHDA-lesioned group receiving i.p. vehicle (Figure 7C,D).

#### 3.3.3. The Binding of [^3^H]nisoxetine to NET

Unilateral administration of 6-OHDA into the left MFB did not affect [^3^H]nisoxetine binding to NET in the lesioned core and shell compared to the corresponding NAcc subregions in the sham-operated group. Furthermore, such treatment with 6-OHDA also did not evoke a significant up-regulation of [^3^H]nisoxetine binding to NET in the intact right core of the 6-OHDA group receiving i.p. vehicle; however, in the latter group, an increasing trend in the NET binding was observed when compared to the corresponding right core in the sham-operated group (Figure 7E).

A two-way ANOVA performed for [^3^H]nisoxetine binding to NET in the lesioned and intact core, as well as in the lesioned shell, revealed a lack of treatment effects of AMI or L-DOPA, but a significant interaction between these drugs (Figure 7E,E’,F,F’). Post hoc comparisons of the examined groups showed that in the group receiving AMI in combination with L-DOPA, NET binding in the core on both sides and in the lesioned shell increased.

#### 3.3.4. Comparative Analysis of Radioligand Binding to Monoamine Transporters in the NAcc in 6-OHDA-Lesioned Rats Treated with AMI and L-DOPA

The effects of a unilateral lesion to the noradrenergic, dopaminergic, and serotonergic systems induced by 6-OHDA injection into the left MFB, as well as of chronic treatment with AMI and L-DOPA, alone or in combination, on radioligand binding to monoamine transporters in the core and shell of the NAcc are summarized in Table 5.

6-OHDA injection into the left MFB caused a drastic decline in DAT binding in the left core and shell of the NAcc but did not change the NET and SERT bindings in the examined NAcc subregions on this side. On the other hand, in the intact right core and shell, a significant increase in DAT and SERT binding was observed following injection of 6-OHDA into the left MFB.

In the lesioned and intact core, as well as in the lesioned shell, both AMI and L-DOPA decreased NET binding, while the combination of AMI+L-DOPA reversed this effect due to drug interaction. In the intact shell, the examined drugs did not change NET binding.

Chronic treatment with AMI did not alter the drastic decrease in DAT binding evoked by 6-OHDA in the lesioned core and shell. However, on the intact side, AMI decreased DAT binding in groups receiving this drug alone and in combination with L-DOPA, both in the core and shell. In the lesioned shell, chronic administration of L-DOPA alone or the combination of AMI+L-DOPA did not change the decrease in DAT binding evoked by 6-OHDA, in contrast to a slight increase in DAT binding in these groups in the lesioned core. On the intact side of the core and shell, L-DOPA alone or the combination of AMI+L-DOPA decreased DAT binding.

Chronic treatment with Ami, L-DOPA, and AMI+L-DOPA decreased SERT binding in the lesioned and intact core and shell. Furthermore, in the lesioned and intact core, as well as in the intact shell, the interaction of AMI and L-DOPA contributes to a decrease in SERT binding.

### 3.4. The Binding of the Radioligand to SERT and NET in the Prefrontal Cortex

**Figure 8:**
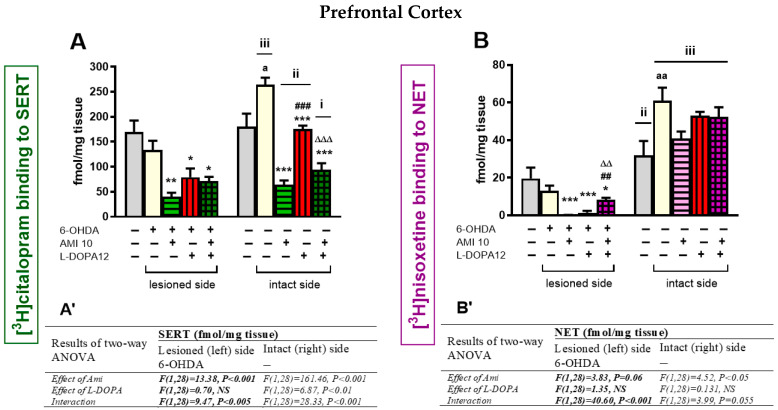
The effects of chronic (21 days) administration of AMI (10 mg/kg i.p.) and L-DOPA (12 mg/kg i.p.), alone and in combination, on the binding of [^3^H]citalopram to SERT (**A**), and [^3^H]nisoxetine to NET (**B**) in the prefrontal cortex on the lesioned and intact sides, determined 1 h after the last drug doses. Data are presented as the mean ± SEM; n = 8 rats per group. Statistical analysis of the SERT and NET binding was performed using a two-way ANOVA, separately for the lesioned and intact sides of the PFC in four groups of rats receiving 6-OHDA unilaterally into the left MFB and the tested drugs i.p. The F values for these analyses are presented in the tables (**A’**,**B’**) below Figure 8A,B. Symbols indicate significance of differences according to the Newman–Keuls post hoc test: * *p* < 0.05, ** *p* < 0.01, *** *p* < 0.001 vs. 6-OHDA+veh-; ^##^ *p* < 0.01; ^###^ *p* < 0.001 vs. 6-OHDA+AMI-; ^ΔΔ^ *p* < 0.01, ^ΔΔΔ^ *p* < 0.001 vs. 6-OHDA+L-DOPA-treated group. The letter “i” indicates statistically significant differences estimated by Student’s *t*-test for dependent samples: ^i^ *p* < 0.05, ^ii^ *p* < 0.01, ^iii^ *p* < 0.001 vs. the corresponding group on the lesioned side. The letter “a” indicates statistically significant differences estimated by Student’s *t*-test for independent samples: ^a^ *p* < 0.05, ^aa^ *p* < 0.01 vs. the sham-operated group.

Due to very low DAT levels in the PFC, the binding of [^3^H]GBR 12,935 to this transporter was not analyzed in this brain structure after chronic administration of the studied drugs. Therefore, in the PFC, only SERT and NET binding levels were examined (Figure 8).

#### 3.4.1. The Binding of [^3^H]citalopram to SERT

Unilateral administration of 6-OHDA into the left MFB did not affect [^3^H]citalopram binding to SERT on the lesioned left side of the PFC compared to the left side of the PFC in the sham-operated group (Figure 8A). However, such administration of 6-OHDA evoked up-regulation of [^3^H]citalopram binding to SERT in the intact PFC compared to the intact side in the sham-operated group (t = −2.847, df = 14, *p* < 0.05).

A two-way ANOVA performed for the [^3^H]citalopram binding to SERT in the lesioned PFC revealed a treatment effect of AMI, no treatment effect of L-DOPA, and a significant interaction between the tested drugs (Figure 8A). The same analysis for SERT binding in the intact PFC showed both significant treatment effects of AMI and L-DOPA, as well as a significant interaction between the examined drugs (Figure 8A). Post hoc comparisons showed that administration of AMI alone or in combination with L-DOPA equally reduced [^3^H]citalopram binding to SERT compared to the 6-OHDA-lesioned group receiving i.p. vehicle. L-DOPA administration alone also significantly reduced [^3^H]citalopram binding in the lesioned PFC; however, on the intact side, the decrease in the SERT binding was smaller than that induced by AMI when compared to the 6-OHDA-lesioned group receiving i.p. vehicle (Figure 8A).

#### 3.4.2. The Binding of [^3^H]nisoxetine to NET

Unilateral administration of 6-OHDA into the left MFB did not affect [^3^H]nisoxetine binding to NET in the lesioned left side of the PFC compared to the corresponding left side in the sham-operated group (Figure 8B). However, such administration of 6-OHDA evoked up-regulation of [^3^H]nisoxetine binding to NET in the intact right PFC compared to the intact right side in the sham-operated group (t = −2.790, df = 14, *p* < 0.05).

A two-way ANOVA performed for the [^3^H]nisoxetine binding to NET in the lesioned PFC revealed a lack of treatment effects of AMI or L-DOPA, but a significant interaction between the tested drugs (Figure 8B). Post hoc comparisons showed that treatment with AMI or L-DOPA alone significantly decreased NET binding in the lesioned PFC, but their combined administration increased this binding in the lesioned PFC.

A two-way ANOVA performed for the [^3^H]nisoxetine binding to NET in the intact PFC showed a significant treatment effect of AMI, no treatment effect of L-DOPA, and an interaction between the tested drugs, which approached statistical significance. AMI administered alone significantly decreased NET binding but the AMI+L-DOPA combination increased it to the level observed in the group receiving L-DOPA alone.

#### 3.4.3. Comparative Analysis of the Radioligand Binding to Monoamine Transporters in the Prefrontal Cortex

Table 6 summarizes the effects of chronic treatment with AMI and L-DOPA, alone or in combination, on the binding of [^3^H]citalopram to SERT and [^3^H]nisoxetine to NET in the PFC of unilaterally 6-OHDA-lesioned rats.

Similarly to the core and shell of the NAcc, 6-OHDA injection into the left MFB induced the up-regulation of the SERT binding in the intact right PFC. However, in contrast to the core and shell of the NAcc, such administration of 6-OHDA also evoked up-regulation of the NET binding in the intact right PFC.

Chronic treatment with AMI alone strongly decreased SERT binding in the lesioned and intact PFC. The decrease in this binding induced by L-DOPA was less pronounced on both sides of the PFC. Moreover, the interaction of AMI and L-DOPA led to decreased SERT binding in the lesioned and intact PFC.

Chronic treatment with AMI alone also strongly decreased NET binding in the lesioned and intact PFC. In the lesioned left PFC, L-DOPA administered alone significantly reduced [^3^H]nisoxetine binding to NET, but acting in combination with AMI, it increased this binding. Chronic treatment with AMI alone also strongly decreased NET binding in the intact PFC, but its combined administration with L-DOPA increased this binding.

### 3.5. The Binding of the [^3^H]citalopram to SERT in the Chosen Region of the Hippocampus

**Figure 9:**
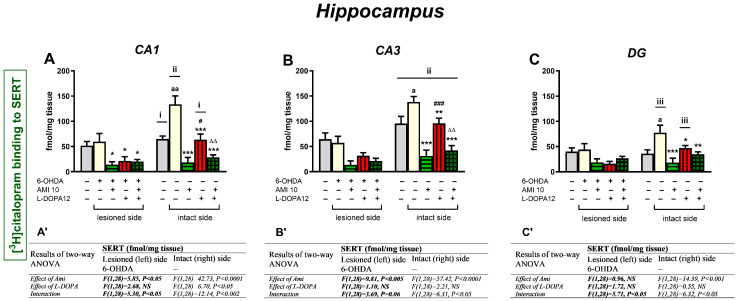
The effects of chronic (21 days) administration of AMI (10 mg/kg i.p.) and L-DOPA (12 mg/kg i.p.), alone and in combination, on the binding of [^3^H] citalopram to SERT in the CA1, CA3, and DG regions of HIP on the lesioned and intact sides, determined 1 h after the last drug doses. Data are presented as the mean ± SEM; n = 8 rats per group. Statistical analysis of the SERT binding was performed using a two-way ANOVA, separately for the lesioned and intact sides of the CA1, CA3, and DG regions of the HIP in four groups of rats receiving 6-OHDA unilaterally into the left MFB and the tested drugs i.p. The F values for these analyses are presented in Tables (**A’**–**C’**) below (**A**–**C**). Symbols indicate significance of differences according to the Newman–Keuls post hoc test: * *p* < 0.05, ** *p* < 0.01, *** *p* < 0.001 vs. 6-OHDA+veh-; ^#^ *p* < 0.05; ^###^ *p* < 0.001 vs. 6-OHDA+AMI-; ^ΔΔ^ *p* < 0.01 vs. 6-OHDA+L-DOPA-treated group. The letter “i” indicates statistically significant differences estimated by Student’s *t*-test for dependent samples: ^i^ *p* < 0.05, ^ii^ *p* < 0.01, ^iii^ *p* < 0.001 vs. the corresponding group on the lesioned side. The letter “a” indicates statistically significant differences estimated by Student’s *t*-test for independent samples: ^a^ *p* < 0.05, ^aa^ *p* < 0.01 vs. the sham-operated group.

Quantitative analysis of [^3^H]citalopram binding to SERT in three regions of the hippocampus (HIP), i.e., in the CA1, CA3, and DG, was performed 1 h after the last chronic doses of the studied drugs (Figure 9).

Like in the lesioned left core and shell of the NAcc and PFC, unilateral administration of 6-OHDA into the left MFB did not affect [^3^H]citalopram binding to SERT in the left CA1, CA3, and DG regions of the HIP compared to the corresponding HIP regions in the sham-operated group (Figure 8A–C). However, such administration of 6-OHDA evoked up-regulation of [^3^H]citalopram binding to SERT in the intact right regions of HIP (Figure 8A–C) compared to the corresponding HIP regions in the sham-operated group (for right CA1, t = −3.840, df = 14, *p* < 0.01; for right CA3, t = 2.410, df = 14, *p* < 0.05; for right DG, t = −2.467, df = 14, *p* < 0.05).

A two-way ANOVA performed for the [^3^H]citalopram binding to SERT in the lesioned left CA1 region of the HIP revealed a treatment effect of AMI, no treatment effect of L-DOPA, and a significant interaction between the tested drugs (Figure 9A). The same analysis for SERT binding in the intact right CA1 showed both significant treatment effects of AMI and L-DOPA, as well as a significant interaction between the examined drugs (Figure 9A).

Post hoc comparison of the examined groups in the lesioned left CA1 region showed that AMI, L-DOPA, and AMI+L-DOPA almost equally reduced [^3^H]citalopram binding to SERT compared to the 6-OHDA-lesioned group receiving i.p. vehicle. Analogical comparison of the examined groups in the intact right CA1 region showed that AMI alone or the combination of AMI+L-DOPA drastically decreased [^3^H]citalopram binding to SERT, whereas the decrease in the SERT binding induced by L-DOPA alone was much smaller compared to the 6-OHDA-lesioned group receiving i.p. vehicle (Figure 9A).

A two-way ANOVA performed for the [^3^H]citalopram binding to SERT in the CA3 and DG regions of the HIP, and the further post hoc comparisons of the tested groups, showed similar effects of AMI and L-DOPA, alone or in combination, on SERT binding on the lesioned left and intact right sides of these HIP regions (Figure 9B,B’,C,C’).

#### Comparison of SERT Binding in the CA1, CA3, and DG Regions of the Hippocampus in 6-OHDA-Lesioned Rats Treated with AMI and L-DOPA

Table 7 summarizes the effects of chronic treatment with AMI and L-DOPA, administered alone or in combination, on the [^3^H]citalopram binding to SERT in the CA1, CA3, and DG regions of the HIP of unilaterally 6-OHDA-lesioned rats.

6-OHDA injection into the left MFB did not change the SERT bindings in CA1, CA3, and DG regions of the HIP on this brain side. However, such administration of 6-OHDA, like in the core and shell of the NAcc and PFC, induced the up-regulation of the SERT binding in the examined regions of the HIP on the intact right side.

In general, chronic treatment with AMI alone evoked the most pronounced decreases in SERT binding in the studied regions of the HIP on the lesioned and intact sides. The decreases in SERT binding evoked by L-DOPA were smaller in the CA1, CA3, and DG regions of the HIP, especially on the intact right side. Furthermore, as in the core and shell of the NAcc and the PFC, the interaction of AMI and L-DOPA played a particular role in reducing SERT binding in the intact right regions of the HIP.

## 4. Discussion

In the present study, a comprehensive analysis of the monoamine metabolism (DA, 5-HT, NA) and radioligand binding to monoamine transporters AT, SERT and NET was performed in the limbic brain structures (NAcc, PFC, HIP) of unilaterally 6-OHDA-lesioned rats treated chronically (21 days) with AMI and L-DOPA, either alone or in combination, in the context of the core symptom of depression, namely anhedonia, which was assessed using the sucrose preference test. This study is a continuation of our previous one, in which the same parameters were analyzed in the STR and SN in the context of motor performance assessed as rotational behavior [43]. Although the interpretation of experimental data obtained in rat motor structures in the context of improved motor function was not straightforward, interpreting analogous changes in limbic structures in relation to the treatment of depression comorbid with motor symptoms is an even more difficult challenge.

Depression is characterized by both emotional and cognitive symptoms, including deficits in attention, working, and episodic memory [58,59]. In the current diagnostic manual, depression in patients is diagnosed based on low mood, anhedonia, and accompanying physical symptoms, such as reduced energy. In animal models of PD, depressive-like behavior, particularly anhedonia, is identified by means of the sucrose preference test [40,42,50]. Clinical and preclinical studies on the neural basis of depressive symptoms have demonstrated abnormalities in the activity of mesocorticolimbic reward circuits, including the VTA, NAcc, HIP, and PFC. At the biochemical level, it is generally accepted that the main cause of major depression is related to the functional impairment of monoaminergic transmission in the limbic and cortical structures of the brain. In Parkinson’s disease, disturbances in monoaminergic transmission, resulting from the progressive neurodegenerative processes, underlie both depression and impaired motor function.

As shown in our previous study, unilateral administration of 6-OHDA into the MFB in rats [40] significantly reduced sucrose solution intake assessed 3 weeks after the lesion, compared with that in the sham-operated control group. At the biochemical level, in these rats, a drastic decrease in NA and DA, as well as a moderate decrease in 5-HT (by about 50%) concentrations, were observed in the PFC, HIP, and STR, including the NAcc, on the lesioned side [40].

The results of our present study show that chronic administration of L-DOPA alone or the combination of AMI+L-DOPA to the unilateral 6-OHDA-lesioned rats improved the intake of sucrose solution compared to the worsened intake of sucrose in the group receiving AMI alone. These effects suggest that L-DOPA plays a key role in reversing anhedonia in the above-described PD model. The increases in sucrose intake were accompanied by increases in the concentrations of L-DOPA-derived DA in the lesioned and intact PFC and HIP. In these brain structures, rats treated with AMI+L-DOPA showed significantly greater increases in L-DOPA-derived DA concentration than those receiving L-DOPA alone. Furthermore, the demonstration of a significant biochemical interaction between AMI and L-DOPA, reflected by the increased concentration of L-DOPA-derived DA, indicates that both drugs modulate DA levels in the PFC and HIP. It is intriguing, however, that rats receiving chronic AMI in combination with L-DOPA experienced remission of anhedonia, whereas chronic administration of AMI alone did not produce such an effect. To clarify this issue, it is necessary to consider the effects of chronic combined treatment with AMI+L-DOPA on the concentration of L-DOPA-derived DA compared to the chronic effect of AMI alone on the concentration of endogenous DA in the PFC, HIP, and NAcc on both sides of the brain.

At physiological conditions, endogenous L-DOPA is metabolized to DA in a reaction catalyzed by aromatic L-amino acid decarboxylase (AADC), exclusively in the dopaminergic neurons. However, in PD and its animal models, due to degeneration of dopaminergic pathways, the conversion of exogenous L-DOPA to DA occurs primarily in serotonergic neurons that also exhibit AADC activity [45,60,61,62]. Therefore, it seems clear that, in our study, exogenous L-DOPA was converted to DA in the serotonergic terminals, in the lesioned PFC, HIP, and NAcc, in the groups receiving this drug, and then it was released as a false neurotransmitter, increasing the extracellular functional pool of DA responsible for behavioral effects. In the intact PFC and NAcc, the conversion of exogenous L-DOPA to DA takes place in the dopaminergic terminals. However, in the intact HIP after administration of L-DOPA alone or in combination with AMI, very high increases in DA levels suggest that the conversion of L-DOPA to DA occurs not only in a few dopaminergic projection terminals from the SN and VTA, but also in serotonergic terminals projecting from the RN. In rats chronically treated with AMI alone, endogenous DA synthesis was virtually absent on the lesioned side of the PFC, HIP, and NAcc due to the loss of most dopaminergic terminals; it occurred only in dopaminergic terminals on the intact side of these brain structures.

This bilateral comparative analysis of DA concentration in the examined limbic structures clearly indicates that the total concentration of this neurotransmitter in the brain of rats chronically treated only with AMI was significantly lower than in rats receiving L-DOPA alone or in combination with AMI, which is of fundamental importance for DA-mediated psychiatric functions. These differences in tissue DA concentrations on both sides of the examined brain structures may explain the severity of anhedonia in the group receiving AMI monotherapy compared to its remission in the group receiving AMI in combination with L-DOPA. Hence, the result obtained in the present study seems to indicate that L-DOPA may be effective in reversing some symptoms of depression, especially anhedonia. This suggestion is consistent with some clinical studies showing a modest improvement in mood following L-DOPA administration [63,64,65], although others have failed to confirm this effect [66,67]. The reason for this discrepancy is unknown, but it appears to be due to differences in the depression symptoms examined, as not all of them respond to L-DOPA treatment. Furthermore, it cannot be ruled out that the dose of L-DOPA may play a significant role in modulating depressive symptoms. In patients with Parkinson’s disease, a significant positive correlation between L-DOPA doses and the level of depressive symptoms has been described [68], suggesting that higher doses of this drug correlate with the worsening of the depressive state. Interestingly, in healthy volunteers, L-DOPA did not exert a positive effect on mood [69], unlike psychostimulants, which strongly and reliably improved it.

Furthermore, L-DOPA, as the only drug able to complete the DA deficiency, is most effective in the treatment of impaired motor functions in PD [70]. As described in our previous study [43], more distinct increases in the content of L-DOPA-derived DA were also observed in the STR and SN in 6-OHDA-lesioned rats treated chronically with AMI+L-DOPA than in rats chronically administered L-DOPA alone. This significantly greater increase in L-DOPA-derived DA concentration following combined administration of the tested drugs was accompanied by a greater improvement in motor function than after L-DOPA alone, as assessed by rotational behavior. The above-described results indicate that L-DOPA at a dose of 12 mg/kg plays a key role not only in improving motor function but also in remitting anhedonia in the unilateral 6-OHDA-induced model of Parkinson’s disease.

The mechanism underlying the significantly greater increase in L-DOPA-derived DA concentration in the presence of chronically administered AMI, compared to chronic exposure to L-DOPA alone, remains an enigma. Therefore, taking into account that AMI has significant affinity for α1-adrenoreceptors (IC50 = 69 nM) [71], it seems reasonable to assume that antagonistic action of this drug at these receptors, via improving cerebral blood flow, may contribute to a greater increase in L-DOPA bioavailability for the rat brain compared to that observed after administration of L-DOPA alone.

On the other hand, considering other potential mechanisms underlying the biochemical interaction of AMI and L-DOPA in the examined limbic brain structures, it should be noted that in rats with unilateral 6-OHDA-induced lesion of dopaminergic pathways, systemic administration of L-DOPA evokes hypersensitivity of postsynaptic DA D1 and D2 receptors in the target structures [41,72]. AMI, in turn, exhibits a significant affinity for DA D1 receptors (IC50 = 81 nM) and acts as an agonist [71]. Chronic administration of AMI to rats with a unilateral 6-OHDA-induced lesion of the nigrostriatal dopaminergic pathway does not appear to sensitize these receptors, because chronic administration of this drug did not produce contralateral rotations, as shown in our previous study [43]. Regarding the effect of AMI on anhedonia in the present study, chronic treatment of 6-OHDA-lesioned rats with this drug worsened the ability to feel pleasure compared to both the sham-operated rats and 6-OHDA-lesioned rats receiving L-DOPA. However, it was reported that AMI facilitated DA-receptor-mediated effects, such as potentiation of the apomorphine-induced gnawing behavior in mice [71]. That study suggested that AMI, by acting on sensitized postsynaptic dopamine receptors, might potentiate their activity. In line with the latter observation, in our previous study, the effect of chronic combined administration of AMI+L-DOPA on rotation behavior was more pronounced than that observed after chronic L-DOPA alone. We can, therefore, speculate that under combined treatment with AMI+L-DOPA, the slowly developing sensitization of postsynaptic DA D1 receptors induced by L-DOPA in the limbic brain structures may be enhanced by AMI, ultimately increasing sucrose intake more significantly than that mediated by L-DOPA alone.

According to the monoaminergic hypothesis, major depression is a result of an abnormal 5-HT and/or NA metabolism in the brain. In suicide victims and depressive patients who died of natural causes, the reduced levels of 5-HT and 5-HIAA were found in the brain stem [73,74] and in the cerebrospinal fluid [75]. In PD, decreased NA and 5-HT concentrations in limbic brain structures resulting from degeneration of ascending noradrenergic and serotonergic pathways may be the primary cause of depressive symptoms [14,15,76,77].

Therefore, the remainder of the discussion will be devoted to analyzing the changes in serotonergic and noradrenergic transmission in the limbic structures of the rat brain (PFC, NAcc, and HIP) following chronic administration of AMI and L-DOPA, alone or in combination, to unilaterally 6-OHDA-lesioned rats. It is worth mentioning that AMI is one of the first tricyclic antidepressants used in clinical practice, which is still administered to a large number of patients with depression. This drug is metabolized to nortriptyline, which has a relatively long half-life [78,79], and as an active metabolite, and it also exhibits therapeutic activity [80]. AMI is a dual 5-HT/NA reuptake inhibitor with similar in vitro reuptake-inhibitory potency for 5-HT and NA (IC50 = 39 and 24 nM, respectively) [81]. Thus, by blocking the reuptake of 5-HT and NA, AMI may increase the extracellular concentrations of these neurotransmitters [78,79], prolonging their action on the postsynaptic receptors that influence specific functions.

The PFC manages many functions involved in the modulation of behavior and thinking, influencing the formation of deliberate, purposeful actions [82,83]. Both DA and NA exert a strong effect on the proper functioning of this brain structure. Optimal functioning of the PFC requires moderate levels of DA and NA; too-low or too-high levels impair its functioning [83,84]. DA is involved in various psychological processes, such as learning, reward, motivation, emotion, and cognition [85,86,87]. Working memory processes in the PFC are modulated by mesocortical DA transmission, which primarily is conveyed via D1 receptors [84,88]. DA deficiency in the PFC and thus, insufficient DA D1 stimulation, impair working memory. In the PFC, due to low expression of DAT [89] and high expression of NET [90,91], the uptake of extracellular DA is mainly mediated by NET [92,93,94,95,96]. Under physiological conditions in the PFC, extracellular DA competes with extracellular NA for NET, and the pools of both neurotransmitters are maintained at the level that guarantees the proper functioning of this structure.

In the present study, due to the unilateral 6-OHDA-induced degeneration of the ascending noradrenergic and dopaminergic pathways from the LC and VTA, drastic decreases in NA and DA concentrations were observed in the lesioned left PFC. Due to the low expression of DAT in the PFC, an autoradiographic analysis of [^3^H]GBR 12,909 binding to DAT was not performed in this brain structure. However, analysis of [^3^H]nisoxetine binding to NET showed a potent increase in NET binding in the intact PFC compared with a drastic decrease in this binding on the lesioned side. The low level of [^3^H]nisoxetine binding to the NET on the lesioned side suggests a loss of these binding sites due to degeneration, while the increased level of [^3^H]nisoxetine binding to the NET on the intact side indicates a compensatory increase in the number of these sites. Because extracellular DA as well as NA are taken up by the NET, this increase in the number of binding sites on the intact side could be considered a compensatory mechanism to regulate extracellular concentrations of these monoamines to maintain proper functioning of the intact side of the PFC. These changes in [^3^H]nisoxetine binding to NET were differently modulated by the tested drugs in the lesioned and intact PFC.

In the intact PFC, the tested drugs increased DA and NA concentrations to varying degrees. However, these changes in NA and DA concentrations on the intact side did not fully correspond to changes in the [^3^H]nisoxetine binding to NET in the above-mentioned groups. In the group receiving AMI alone, a pronounced decrease in [^3^H]nisoxetine binding to NET at noradrenergic terminals indicates a significant inhibition of the function of this transporter by the antidepressant. However, in the group treated with the combination of AMI+L-DOPA, the interaction between these drugs increased the binding of [^3^H]nisoxetine to NET. In fact, the level of NET binding in this group remained as high as in the group receiving L-DOPA alone. In general, inhibition of NET function leads to an increase in extracellular NA concentration, a reduction in DA uptake, and, consequently, to an increase in extracellular DA concentration. Such an effect was observed in the AMI-treated group. In the group receiving the combination of AMI+L-DOPA, the increase in DA content was greater than in the group administered L-DOPA alone. Furthermore, in the AMI+L-DOPA-treated group, an increase in DA concentration was accompanied by an enhancement of NA content, although slightly smaller than in the group receiving AMI alone. However, in the group receiving only L-DOPA, despite a significant increase in DA concentration, the NA content did not change.

The above-presented effects suggest, on the one hand, that the high level of NET in the groups receiving AMI+L-DOPA or L-DOPA alone may participate in the uptake of the excess amount of L-DOPA-derived DA. On the other hand, such high levels of NET may also enhance NA reuptake, reducing the extracellular NA concentration. In our study, the lack of change in NA concentration in the group treated with L-DOPA alone suggests that higher doses of this drug may increase the predominance of extracellular DA over extracellular NA, ultimately leading to an imbalance between the extracellular DA and NA pools. In contrast, in the group receiving AMI+L-DOPA, the increase in DA concentration was accompanied by an increase in NA concentration, which indicates that the balance between the functional pools of these neurotransmitters is maintained.

Similar changes in DA concentrations and [^3^H]nisoxetine binding to NET in the groups receiving the tested drugs were observed in the lesioned PFC. However, changes in the expression of these parameters cannot be analyzed in relation to NA concentration because of its very low level resulting from the loss of left-sided noradrenergic projections.

The above-presented comparative analysis of DA and NA concentrations in relation to [^3^H]nisoxetine binding to the NET in both the lesioned and intact PFC suggests that NET expression in the PFC may be modulated depending on the DA concentration in this brain structure. These results indicate that such modulation occurs both in the 6-OHDA-treated control group receiving i.p. vehicle, and in the 6-OHDA-treated groups receiving the tested drugs i.p. Therefore, it can be assumed that the modulation of NET expression in the PFC is crucial for maintaining the balance between the extracellular functional pools of DA and NA, so that this brain structure can function, either less or more properly, in pathological conditions and during therapy.

Using multisite microdialysis in unilaterally 6-OHDA-lesioned rats, it was directly confirmed that L-DOPA administration at a dose of 12 mg/kg, like in our study, evoked DA release in the PFC and HIP on the lesioned side [45,97]. Furthermore, NET blockers desipramine and reboxetine potentiated L-DOPA effects in these brain structures [98]. Since AMI can inhibit NET, it is reasonable to assume that it may also potentiate the effects of L-DOPA. In our study, only the total tissue concentration of DA and its metabolites was determined in the PFC and HIP. The tissue DA content reflects both the intracellular pool of this neurotransmitter and its extracellular functional fraction. The release of L-DOPA-derived DA, or rather its extracellular concentration in rats treated with the studied drugs, was assessed indirectly, based on the concentrations of DA metabolites. This analysis showed that, in the PFC and HIP of rats treated with AMI+L-DOPA, the concentrations of DOPAC, 3-MT and HVA were higher than in the L-DOPA-treated group. In general, L-DOPA accelerates catabolism of L-DOPA-derived DA assessed as DOPAC/DA, 3-MT/DA and HVA/DA metabolic ratios. However, in rats receiving AMI+L-DOPA, the values of these parameters were lower than in the group treated with L-DOPA alone, suggesting attenuation DA catabolism in the presence of AMI. Therefore, the attenuation of DA catabolism may indicate an increase in the extracellular pool of this transmitter in the examined limbic structures.

The NAcc is one of the basal ganglia of the brain, which plays a key role in action selection by integrating cognitive and affective information processed by certain regions of the PFC to enhance the effectiveness of appetitively or aversively motivated behaviors [99]. This brain structure is the site of signal reception from the ascending dopaminergic projections from the VTA. Functionally, the NAcc, as a part of the mesolimbic dopaminergic pathway, plays a key role in the cognitive processing of motivation, pleasure, reward, and motor program encoding. Due to anatomical and functional differences, the NAcc is divided into two subregions, i.e., the core and the shell [99]. In terms of reward-seeking and pleasure-seeking behaviors, the core of the NAcc is thought to mediate action-stimulating behaviors, while the shell suppresses behavioral patterns that may interfere with goal pursuit [99]. The core promotes responses to stimuli signaling the availability of a reward, and its activity ensures the organism’s pursuit of the location where the reward can be obtained. In contrast, the shell suppresses behaviors directed toward irrelevant, unrewarded, or less-preferred outcomes, focusing the organism on seeking and obtaining reward.

In the present study, we did not determine DA concentration in the core and shell of the NAcc in any of the studied groups. However, in the previous study [43], DA concentration was assayed in the striatum, which also included NAcc. In the latter study [43], AMI administered in combination with L-DOPA induced a significantly greater increase in the tissue concentration of L-DOPA-derived DA in the STR than L-DOPA alone. Depressive behaviors are related to a lack of motivation, while the DA action in the NAcc is associated with the stimulation of motivational behaviors [100]. Therefore, in our study, it is reasonable to assume that the relatively large increase in DA concentration in the NAcc of rats receiving AMI in combination with L-DOPA could increase motivation to seek a sucrose solution as a reward.

The shell subregion of the NAcc receives moderately dense noradrenergic innervation, in contrast to the striatum, which is almost devoid of noradrenergic afferents. In the shell, most of the noradrenergic afferents arise from the nucleus tractus solitarius (NTS; A2 region) and much less from the caudal ventrolateral medulla (CVLM; A1 region) or the LC [101,102]. In the present study, we did not determine NA levels in the core and shell of the NAcc in any of the studied groups. However, we analyzed [^3^H]nisoxetine binding to NET in all these groups. Interestingly, the levels of NET binding in the lesioned and intact core, as well as in the lesioned shell, in the group receiving AMI+L-DOPA, were markedly higher than in the corresponding sides in the groups receiving AMI or L-DOPA alone. As to the [^3^H]GBR12935 binding to DAT, 6-OHDA administration into the left MFB resulted in degeneration of the dopaminergic pathways innervating the NAcc, and consequently, in a loss of DAT binding sites in the core and shell on the lesioned side. Under physiological conditions, high DAT expression in the NAcc contributes to the DA reuptake. However, in the DA-denervated NAcc, NET may play this role, as in brain structures with low DAT expression, such as the PFC. Thus, in the lesioned core and shell, the high expression of NET in the groups receiving AMI+L-DOPA may participate in the reuptake of the excess amount of L-DOPA-derived DA. In the intact shell, both DAT and NET may contribute to maintaining the balance between the extracellular pool of DA and NA.

Summing up, from the perspective of maintaining the balance between extracellular DA and NA pools in the PFC and NAcc, in PD therapy, the combined administration of AMI+L-DOPA appears to be more beneficial than L-DOPA alone.

The hippocampus is a limbic brain structure critical for cognitive and emotional regulation. It receives few dopaminergic projections from the SNc and VTA [103,104,105,106]. The concentration of DA in the HIP is very low, many times lower than in the PFC [40], but it is of great functional importance, as it modulates hippocampal long-term potentiation [106,107]. As shown in our previous studies [40,43], unilateral degeneration of dopaminergic neurons in the SNc, induced by 6-OHDA injection into the left MFB [40,43], resulted in a loss of hippocampal DA [40]. Hippocampal long-term potentiation represents an experimental model for the synaptic changes underlying learning and memory processes [108,109]. In the 6-OHDA-induced model of PD, alterations in hippocampal long-term potentiation were associated with impaired DA transmission and deficits in HIP-dependent learning processes [108,109]. L-DOPA restored the hippocampal long-term potentiation impaired by 6-OHDA, via stimulation of dopamine D1/D5 receptors, and ameliorated cognitive deficits induced by this neurotoxin [108].

In our study, both L-DOPA alone and the combination of AMI+L-DOPA significantly increased tissue DA concentration in the lesioned and intact HIP. In the HIP, DAT expression is low [110,111], and NET is the main factor regulating extracellular DA concentration [112,113,114], as in the PFC. In our study, we cannot assess the role of NET in regulating the extracellular DA level in the lesioned and intact HIP, as [^3^H]nisoxetine binding to NET was not investigated. However, an analysis of DA catabolism, described above, indicates that an increase in the tissue DA concentrations also affects its functional extracellular pool, playing a crucial role in learning and memory.

Regarding the impact of L-DOPA administered alone or in combination with AMI on depressive symptoms, it is worth noting that in brain structures with DA denervation, such as the PFC and HIP in our study, L-DOPA was converted to DA in serotonergic terminals [45,60,61,62]. In these terminals, L-DOPA-derived DA competes with 5-HT for the vesicular monoamine transporter VMAT2 to enter exocytotic vesicles [62,97], which finally leads to DA accumulation in 5-HT vesicles, probably at the expense of endogenous 5-HT. In our study, chronic treatment with L-DOPA alone significantly decreased the tissue 5-HT content in the lesioned PFC and HIP. However, combined administration of AMI+L-DOPA increased it (see interactions of AMI and L-DOPA, Figure 5A’,B’). Moreover, a decreased value of 5HIAA/5-HT metabolic ratio found in the lesioned PFC in rats treated with AMI+L-DOPA indicates that the extracellular pool of 5-HT was also increased. The above-described effect in rats treated with AMI+L-DOPA is consistent with the study by Navailles et al. [98], who found that L-DOPA enhanced the extracellular 5-HT level only in the presence of NET blockers and in those brain regions where DA levels were also enhanced. AMI is a dual inhibitor of SERT and NET; therefore, the increase in extracellular 5-HT levels in rats treated with AMI+L-DOPA on the lesioned side of the PFC may result from the cooperative action of AMI on SERT and NET.

Antidepressants act by rapidly and effectively blocking 5-HT reuptake. However, the clinical effects of these drugs are only apparent after 3 weeks. This means that the therapeutic effect occurs not only through the inhibition of 5-HT reuptake but also through long-term adaptive changes. For a long time, it was thought that chronic treatment with these drugs down-regulated and desensitized presynaptic 5-HT1A receptors in the raphe nuclei, relieving 5-HT neurons from self-inhibition and resulting in the release of 5-HT [115,116]. However, it is suggested that desensitization of SERT also plays an important role in the therapeutic action of antidepressants [117,118].

In our study, an autoradiographic analysis of [^3^H]citalopram binding to SERT performed in rats administered AMI alone or in combination with L-DOPA showed that such treatment strongly decreased the binding of this ligand to SERT in the PFC, NAcc, and in all regions of the HIP on both sides of the brain. As the tested drugs were administered chronically for 21 days, it seems that this decline in SERT binding may result rather from down-regulation of SERT than from blockade of its function by AMI. Administration of L-DOPA alone also reduced [^3^H]citalopram binding to SERT in all these brain structures, only on the lesioned side. In contrast, on the intact side, in the NAcc and HIP, [^3^H]citalopram binding to SERT in the L-DOPA-treated group was almost the same as in the sham-operated control, and only in the intact PFC was it slightly higher than in the control. The reduction in [^3^H]citalopram binding to SERT under the influence of AMI administered alone or in combination indicates a limited capacity for 5-HT reuptake and, therefore, an increase in the functional extracellular pool of this neurotransmitter in all studied limbic structures. These increases in the extracellular 5-HT concentration in limbic structures, especially after combined administration of AMI and L-DOPA, may be important for mitigation of depressive symptoms in PD.

In conclusion, the above-presented interpretation of the behavioral and biochemical data suggests that combined therapy using the antidepressant amitriptyline and the antiparkinsonian drug L-DOPA may be effective in alleviating depressive symptoms in Parkinson’s disease. AMI, as an antidepressant with similar potency in inhibiting 5-HT and NA, may regulate both impaired serotonergic and noradrenergic transmission and modulate L-DOPA-induced dopaminergic transmission.

## 5. Limitation

In the present study, the sucrose preference test was performed only in groups of rats with a unilateral lesion of the monoaminergic systems induced by 6-OHDA injection into the left MFB, which were treated chronically with AMI and L-DOPA, either alone or in combination. However, this study did not include a sham-operated group receiving AMI alone, which would have provided an additional control for the effects of this drug. The lack of such a group is a limitation of this study.

The second limitation of this study is the timing of the sucrose preference test, conducted over 24 h, while monoamine concentrations were determined only at one time point, 1 hour after L-DOPA administration. This means that the measurement of sucrose consumption is a mixed effect covering both the “on” phase, i.e., the effect of L-DOPA, and the “off” phase, i.e., turning off its action.

## 6. Conclusions

The behavioral and biochemical data presented in this study demonstrate that a 21-day combined chronic therapy with the antidepressant drug amitriptyline and the antiparkinsonian drug L-DOPA results in remission of anhedonia, as measured by the sucrose preference test, in the unilateral 6-OHDA-induced rat model of Parkinson’s disease. L-DOPA, the only drug capable of completing DA deficiency, when administered in combination with AMI, more distinctly increased the concentration of this neurotransmitter in the lesioned PFC as well as in the lesioned and intact HIP than L-DOPA administered alone. AMI, as an antidepressant with similar potency in inhibiting 5-HT and NA, may regulate the impaired serotonergic and noradrenergic transmission and modulate L-DOPA-induced dopaminergic transmission.

## Figures and Tables

**Table 1:** The effect of systemic administration of amitriptyline (Ami; 10 mg/kg) or L-DOPA (12 mg/kg), alone and in combination for 3 weeks, on the levels of DA metabolites (DOPAC, 3-MT, HVA) in the prefrontal cortex and hippocampus of unilaterally 6-OHDA-lesioned rats.

Brain Structures	DOPAC (ng/g Tissue)		3-MT (ng/g Tissue)		HVA (ng/g Tissue)	
	Lesioned (Left) Side 6-OHDA	Intact (Right) Side –	Lesioned (Left) Side 6-OHDA	Intact (Right) Side –	Lesioned (Left) Side 6-OHDA	Intact (Right) Side –
***Prefrontal cortex***						
veh	3.4 ± 0.7	151 ± 17 ^iii^	1.7 ± 0.2	20 ± 2 ^iii^	3.7 ± 0.5	81 ± 6 ^iii^
AMI(10)	3.2 ± 0.6	240 ± 21 ^iii^	1.6 ± 0.1	24 ± 5 ^ii^	4.5 ± 0.7	114 ± 6 ^iii^
L-DOPA(12)	108 ± 11 ***^,###^	503 ± 35 ^iii^	7.8 ± 2	41 ± 4 ^iii^	157 ± 16 ***^,###^	447 ± 29 ^iii^
AMI+L-DOPA	259 ± 18 ***^,###,∆∆∆^	699 ± 39 ^iii^	13.4 ± 1.5	42 ± 4 ^iii^	285 ± 24 ***^,###,∆∆∆^	557 ± 35 ^iii^
*Effect of AMI*	*F(1,44) = 31.7, p < 0.001*	*F(1,44) = 16.9, p < 0.001*	*no*	*no*	*F(1,44) = 12.7, p < 0.001*	*F(1,44) = 6.0, p < 0.05*
*Effect of L-DOPA*	*F(1,44) = 180.3, p < 0.001*	*F(1,44) = 136.9, p < 0.001*	*F(1,44) = 34.7, p < 0.001*	*F(1,44) = 22.2, p < 0.001*	*F(1,44) = 143.4, p < 0.001*	*F(1,44) = 196.0, p < 0.001*
*Interaction*	*F(1,44) = 31.9, p < 0.001*	*no*	*no*	*no*	*F(1,44) = 12.4, p < 0.001*	*no*
***Hippocampus***						
veh	5.5 ± 0.8	7.1 ± 0.7	2.4 ± 0.4	3.7 ± 0.8	2.7 ± 0.5	3.5 ± 0.4
AMI(10)	1.6 ± 0.3	3.8 ± 0.3 ^ii^	1.9 ± 0.5	2.1 ± 0.5	2.5 ± 0.6	3.9 ± 0.6 ^ii^
L-DOPA(12)	97 ± 6 ***^,###^	135 ± 11 ***^,###,iii^	4.0 ± 0.8	5.0 ± 1 ^i^	147 ± 9 ***^,###^	168 ± 11 ***^,###,iii^
AMI+L-DOPA	165 ± 12 ***^,###,∆∆∆^	214 ± 14 ***^,###,∆∆∆,iii^	6.2 ± 0.8	7.2 ± 0.9	210 ± 19 ***^,###,∆∆^	245 ± 23 ***^,###,∆∆,iii^
*Effect of AMI*	*F(1,44) = 14.8, p < 0.001*	*F(1,44) = 12.0, p < 0.01*	*no*	*no*	*F(1,44) = 5.3, p < 0.05*	*F(1,44) = 5.4, p < 0.05*
*Effect of L-DOPA*	*F(1,44) = 231.0, p < 0.001*	*F(1,44) = 236.3, p < 0.001*	*F(1,44) = 16.2, p < 0.001*	*F(1,44) = 1138, p < 0.001*	*F(1,44) = 167.0, p < 0.001*	*F(1,44) = 151.8, p < 0.001*
*Interaction*	*F(1,44) = 18.5, p < 0.001*	*F(1,44) = 14.2, p < 0.001*	*no*	*F(1,44) = 3.9, p = 0.053*	*F(1,44) = 5.4, p < 0.05*	*F(1,44) = 5.3, p < 0.05*

The data are presented as the mean ± S.E.M.; the number of rats per group was n = 9–15. Significance of differences in paired Student’s *t*-test: ^i^ *p* < 0.05, ^ii^ *p* < 0.01, ^iii^ *p* < 0.001 vs. the lesioned side of a particular group. Significance of differences between the examined groups within the lesioned and intact side of the PFC or HIP was calculated using a two-way ANOVA, followed, if a significant interaction was found, by the Newman–Keuls post hoc test: *** *p* < 0.001 vs. veh-treated group, ^###^ *p* < 0.001 vs. Ami(10)-treated group, ^∆∆^ *p* < 0.01, ^∆∆∆^ *p* < 0.001 vs. the L-DOPA(12)-treated group of corresponding lesioned or intact sides.

**Table 2:** The effect of systemic administration of amitriptyline (Ami; 10 mg/kg) or L-DOPA (12 mg/kg), alone and in combination for 3 weeks, on the dopamine turnover in the prefrontal cortex and hippocampus of unilaterally 6-OHDA-lesioned rats.

Brain Structures	DOPAC/DA		3-MT/DA		HVA/DA	
	Lesioned (Left) Side 6-OHDA	Intact (Right) Side –	Lesioned (Left) Side 6-OHDA	Intact (Right) Side –	Lesioned (Left) Side 6-OHDA	Intact (Right) Side –
***Prefrontal cortex***						
veh	41 ± 5	20 ± 3 ^ii^	19 ± 2	2.7 ± 0.3 ^iii^	43 ± 5	11 ± 1 ^iii^
AMI(10)	29 ± 2	24 ± 4	22 ± 7	2.2 ± 0.3 ^i^	46 ± 6	11 ± 1 ^iii^
L-DOPA(12)	156 ± 16	49 ± 4 ^iii^	17 ± 7	3.8 ± 0.3	236 ± 33 ***^,###^	44 ± 4 ^iii^
AMI+L-DOPA	105 ± 7	50 ± 5 ^iii^	5.8 ± 1	2.9 ± 0.2 ^i^	118 ± 11 *^,#,∆∆∆^	39 ± 2 ^iii^
*Effect of AMI*	*F(1,44) = 8.6, p < 0.01*	*no*	*no*	*F(1,44) = 6.3, p < 0.02*	*F(1,44) = 7.0, p < 0.02*	*no*
*Effect of L-DOPA*	*F(1,44) = 76.0, p < 0.001*	*F(1,44) = 40.7, p < 0.001*	*no*	*F(1,44) = 10.7, p < 0.001*	*F(1,44) = 38.1, p < 0.001*	*F(1,44) = 124.0, p < 0.001*
*Interaction*	*no*	*no*	*no*	*no*	*F(1,44) = 7.9, p < 0.01*	*no*
***Hippocampus***						
veh	176 ± 24	74 ± 9 ^ii^	89 ± 19	38 ± 10 ^i^	89 ± 16	39 ± 7 ^i^
AMI(10)	42 ± 9 ***	38 ± 5	40 ± 5 **	19 ± 3 *^,ii^	55 ± 10	36 ± 4
L-DOPA(12)	162 ± 8 ^###^	107 ± 7 ^###,iii^	7 ± 1 ***^,#^	3.8 ± 0.7 ***^,ii^	259 ± 26	129 ± 9 ^iii^
AMI+L-DOPA	155 ± 11 ^###^	114 ± 13 *^,###,i^	6 ± 1 ***^,#^	3.6 ± 0.4 ***^,ii^	206 ± 26	114 ± 9 ^iii^
*Effect of AMI*	*F(1,44) = 25.9, p < 0.001*	*F(1,44) = 12.0, p < 0.01*	*F(1,44) = 8.3, p < 0.01*	*F(1,44) = 5.0, p < 0.05*	*no*	*no*
*Effect of L-DOPA*	*F(1,44) = 12.9, p < 0.001*	*F(1,44) = 236.3, p < 0.001*	*F(1,44) = 46.4, p < 0.001*	*F(1,44) = 34.5, p < 0.001*	*F(1,44) = 44.4, p < 0.001*	*F(1,44) = 89.7, p < 0.001*
*Interaction*	*F(1,44) = 21.1, p < 0.001*	*F(1,44) = 14.2, p < 0.001*	*F(1,44) = 8.1, p < 0.01*	*F(1,44) = 4.8, p < 0.05*	*no*	*no*

The data are presented as the mean ± S.E.M.; the number of rats per group was n = 9–15. Significance of differences in paired Student’s *t*-test: ^i^ *p* < 0.05, ^ii^ *p* < 0.01, ^iii^ *p* < 0.001 vs. the lesioned side of a particular group. Significance of differences between the metabolic indices DOPAC/DA, 3-MT/DA and HVA/DA in the lesioned and intact PFC or HIP was calculated using a two-way ANOVA, followed by the Newman–Keuls test, if a significant interaction occurred: * *p* < 0.05, ** *p* < 0.01, *** *p* < 0.001 vs. veh-treated group, ^#^ *p* < 0.05, ^###^ *p* < 0.001 vs. AMI(10)-treated group, ^∆∆∆^ *p* < 0.001 vs. the L-DOPA(12)-treated group of corresponding lesioned or intact sides.

**Table 3:** The effect of systemic administration of amitriptyline (Ami; 10 mg/kg) or L-DOPA (12 mg/kg), alone and in combination for 3 weeks, on the level of 5-HT metabolite (5-HIAA) and on 5-HT turnover assessed as metabolic index of 5-HIAA/5-HT in the prefrontal cortex and hippocampus of unilaterally 6-OHDA-lesioned rats.

Brain Structures	5-HIAA (ng/g Tissue)		5-HIAA/5-HT	
	Lesioned (Left) Side 6-OHDA	Intact (Right) Side –	Lesioned (Left) Side 6-OHDA	Intact (Right) Side –
***Prefrontal cortex***				
veh	142 ± 17	230 ± 9 ^ii^	50 ± 3	43.5 ± 2 ^ii^
AMI(10)	129 ± 15	253 ± 18 ^ii^	86 ± 7	54.2 ± 3 ^iii^
L-DOPA(12)	113 ± 21	287 ± 13 ^iii^	143 ± 20 ***^,#^	72.3 ± 5 ^ii^
AMI+L-DOPA	201 ± 19 *^,#,∆∆^	306 ± 11 ^iii^	102 ± 10 *^,∆^	75.1 ± 6 ^iii^
*Effect of AMI*	*F(1,44) = 3.6, p = 0.06*	no	no	no
*Effect of L-DOPA*	*no*	*F(1,44) = 17.8, <0.001*	*F(1,44) = 15.2, p < 0.001*	*F(1,44) = 22.8, p < 0.001*
*Interaction*	*F(1,44_)_ = 6.5, p < 0.02*	*no*	*F(1,44) = 7.6, p < 0.01*	*no*
***Hippocampus***				
veh	218 ± 30	367 ± 17 ^ii^	160 ± 19	96 ± 4 ^ii^
AMI(10)	165 ± 15	340 ± 17 ^iii^	210 ± 36	122 ± 14 ^i^
L-DOPA(12)	128 ± 8 **	311 ± 11 *^,iii^	366 ± 34	175 ± 22 ^iii^
AMI+L-DOPA	201 ± 19 ^∆^	350 ± 11 ^iii^	311 ± 46	187 ± 26
*Effect of AMI*	*no*	*no*	*no*	*no*
*Effect of L-DOPA*	*no*	*no*	*F(1,44) = 15.2, p < 0.001*	*F(1,44) = 10.7, p < 0.01*
*Interaction*	*F(1,44) = 10.8, p < 0.002*	*F(1,44) = 6.0, p < 0.02*	*no*	*no*

The data are presented as the mean ± S.E.M.; the number of rats per group was n = 9–15. Significance of differences in paired Student’s *t*-test: ^i^ *p* < 0.05, ^ii^ *p* < 0.01, ^iii^ *p* < 0.001 vs. the lesioned side of a particular group. Significance of differences between the examined groups in the PFC and HIP was calculated using a two-way ANOVA, followed by the Newman–Keuls test, if a significant interaction was found: * *p* < 0.05, ** *p* < 0.01, *** *p* < 0.001 vs. vehicle-treated group, ^#^ *p* < 0.05, vs. Ami(10)-treated group, ^∆^ *p* < 0.05, ^∆∆^ *p* < 0.01 vs. the L-DOPA(12)-treated group of corresponding lesioned or intact sides.

**Table 4:** Comparison of the effects of chronic administration of AMI and L-DOPA, alone and in combination, on monoamine concentrations in the prefrontal cortex and hippocampus of unilaterally 6-OHDA-lesioned rats.

Tested Groups	Tissue Monoamine Concentrations					
	*Lesioned Left Side*			*Intact Right Side*		
	NA	DA	5-HT	NA	DA	5-HT
***Prefrontal cortex***						
*6-OHDA+veh*	**⮯⮯**	**⮯⮯**	**⮯**	**⮯**	**cl**	**cl**
*6-OHDA+AMI(10)*	**nc**	**nc**	**nc**	**⮭**	**⮭**	**nc**
*6-OHDA+L-DOPA(12)*	**nc**	**⮭**	**⮯**	**nc**	**⮭**	**nc**
*6-OHDA+AMI+L-DOPA*	**nc**	**⮭⮭**	**⮭**	**⮭**	**⮭⮭**	**nc**
***Hippocampus***						
*6-OHDA+veh*	**⮯⮯**	**⮯⮯**	**⮯**	**⮯**	**cl**	**cl**
*6-OHDA+AMI(10)*	**nc**	**nc**	**nc**	**nc**	**nc**	**nc**
*6-OHDA+L-DOPA(12)*	**nc**	**⮭**	**⮯**	**nc**	**⮭**	**⮯**
*6-OHDA+AMI+L-DOPA*	**nc**	**⮭⮭**	**⮭**	**nc**	**⮭⮭**	**⮯**

Blue arrows indicate decreases in monoamine concentrations induced by 6-OHDA on the lesioned side: **⮯⮯**—strong effect; **⮯**—moderate effect. The green arrow **⮯** indicates a small decline in the NA concentration in the intact side, according to the study by Kamińska et al. [40]. Red arrows indicate the interaction of AMI and L-DOPA: **⮭⮭**—strong effect; **⮭**—moderate effect. Black arrows indicate: **⮭**—moderate increase, **⮭⮭**—strong increase; and **⮯**—moderate decrease in monoamine concentrations induced by AMI and L-DOPA administered alone or in combination. Abbreviations: **nc/nc**—no effect of the tested drugs on monoamine levels on the lesioned or intact side; cl—control level of monoamines.

**Table 5:** The effects of chronic administration of amitriptyline (Ami; 10 mg/kg i.p.) or L-DOPA (12 mg/kg i.p.), alone and in combination, on radioligands binding to NET, DAT and SERT in the lesioned and intact core and shell of nucleus accumbens.

Tested Groups	Radioligand Binding to Monoamine Transporters					
	*Lesioned Left Side*			*Intact Right Side*		
	NET	DAT	SERT	NET	DAT	SERT
***Nucleus accumbens—core***						
*Sham+veh*	**cl**	**cl**	**cl**	**cl**	**cl**	**cl**
*6-OHDA+veh*	**nc**	**⮯⮯**	**nc**	**nc**	**⮭⮭**	**⮭⮭**
*6-OHDA+AMI(10)*	**⮯⮯**	**nc**	**⮯⮯**	**⮯⮯**	**⮯**	**⮯⮯**
*6-OHDA+L-DOPA(12)*	**⮯⮯**	**⮭**	**⮯**	**⮯⮯**	**⮯**	**⮯**
*6-OHDA+AMI+L-DOPA*	**⮭⮭**	**⮭**	**⮯**	**⮭⮭**	**⮯⮯**	**⮯⮯**
***Nucleus accumbens—shell***						
*Sham+veh*	**cl**	**cl**	**cl**	**cl**	**cl**	**cl**
*6-OHDA+veh*	**nc**	**⮯⮯**	**nc**	**nc**	**⮭**	**⮭⮭**
*6-OHDA+AMI(10)*	**⮯**	**nc**	**⮯⮯**	**nc**	**⮯**	**⮯⮯**
*6-OHDA+L-DOPA(12)*	**⮯⮯**	**nc**	**nc**	**nc**	**nc**	**⮯**
*6-OHDA+AMI+L-DOPA*	**⮭**	**nc**	**⮯⮯**	**nc**	**⮯**	**⮯⮯**

Blue arrows indicate decreases in the DAT binding induced by 6-OHDA on the lesioned side: **⮯⮯**—strong effect. Green arrows indicate up-regulation in the DAT and SERT binding on the intact side: **⮭⮭**—strong effect; **⮭**—moderate effect. Red arrows indicate changes in the interaction of AMI and L-DOPA: either the increase: **⮭⮭**—strong effect; **⮭**—moderate effect or the decrease: **⮯⮯**—strong effect, **⮯**—moderate effect. Black arrows indicate either increases: **⮭**—moderate effect or decreases: **⮯**—moderate effect and **⮯⮯**—strong effect in the binding to monoamine transporters induced by AMI and L-DOPA administered alone or in combination. Abbreviations: **nc/nc**—no effect of the tested drugs on radioligand binding on the lesioned or intact side; cl—control level of radioligands binding to monoamine transporters.

**Table 6:** The effects of chronic administration of amitriptyline (Ami; 10 mg/kg i.p.) or L-DOPA (12 mg/kg i.p.), alone and in combination, on radioligands binding to NET and SERT in the lesioned (left) and intact (right) side of the prefrontal cortex.

Tested Groups	Radioligand Binding to Monoamine Transporters			
	*Lesioned Left Side*		*Intact Right Side*	
	NET	SERT	NET	SERT
***Prefrontal cortex***				
*Sham+veh*	**cl**	**cl**	**cl**	**cl**
*6-OHDA+veh*	**nc**	**nc**	**⮭⮭**	**⮭⮭**
*6-OHDA+AMI(10)*	**⮯⮯**	**⮯⮯**	**⮯⮯**	**⮯⮯**
*6-OHDA+L-DOPA(12)*	**⮯⮯**	**⮯**	**nc**	**⮯**
*6-OHDA+AMI+L-DOPA*	**⮭**	**⮯**	**⮭**	**⮯⮯**

Green arrows indicate up-regulation of NET and SERT binding in the intact side: **⮭⮭**—strong effect. Red arrows indicate changes in the interaction of AMI and L-DOPA: either the increase: **⮭**—moderate effect or the decrease: **⮯⮯**—strong effect, **⮯**—moderate effect. Black arrows indicate either the increase: **⮭**—moderate effect or decreases: **⮯**—moderate effect and **⮯⮯**—strong effect in the NET and SERT binding induced by AMI and L-DOPA administered alone or in combination. Abbreviations: **nc**/**nc**—no effect of the tested drugs on the NET and SERT binding on the lesioned or intact side; cl—control level of the NET and SERT binding.

**Table 7:** The effects of chronic administration of amitriptyline (Ami; 10 mg/kg i.p.) or L-DOPA (12 mg/kg i.p.), alone and in combination, on [^3^H] citalopram binding to SERT in the lesioned (left) and intact (right) CA1, CA3, and DG regions of the hippocampus.

Tested Groups	[^3^H]citalopram Binding to SERT	
	*Lesioned Left Side*	*Intact Right Side*
	SERT	SERT
***CA1***		
*Sham+veh*	**cl**	**cl**
*6-OHDA+veh*	**nc**	**⮭⮭**
*6-OHDA+AMI(10)*	**⮯⮯**	**⮯⮯**
*6-OHDA+L-DOPA(12)*	**⮯**	**⮯**
*6-OHDA+AMI+L-DOPA*	**⮯**	**⮯⮯**
***CA3***		
*Sham+veh*	**cl**	**cl**
*6-OHDA+veh*	**nc**	**⮭⮭**
*6-OHDA+AMI(10)*	**⮯⮯**	**⮯⮯**
*6-OHDA+L-DOPA(12)*	**⮯**	**⮯**
*6-OHDA+AMI+L-DOPA*	**⮯**	**⮯⮯**
***DG***		
*Sham+veh*	**cl**	**cl**
*6-OHDA+veh*	**nc**	**⮭⮭**
*6-OHDA+AMI(10)*	**⮯**	**⮯⮯**
*6-OHDA+L-DOPA(12)*	**⮯**	**⮯**
*6-OHDA+AMI+L-DOPA*	**⮯**	**⮯**

Green arrows indicate up-regulation of SERT binding on the intact side: **⮭⮭**—strong effect. Red arrows indicate decreases in the interaction of AMI and L-DOPA: **⮯⮯**—strong effect, **⮯**—moderate effect. Black arrows indicate decreases in the SERT binding induced by AMI and L-DOPA administered alone or in combination: **⮯**—moderate effect and **⮯⮯**—strong effect. Abbreviations: **nc**/**nc**—no effect of the tested drugs on the SERT binding on the lesioned or intact side; cl—control level of the SERT binding.

## Data Availability

Data supporting the reported results are available on request from the corresponding author.
